# Strategies to enhance clinical teaching and learning in undergraduate nursing education: A scoping review

**DOI:** 10.1371/journal.pone.0305789

**Published:** 2025-06-10

**Authors:** Kafui A. Hobenu, Anthonio O. Adefuye, Florence Naab, Champion N. Nyoni

**Affiliations:** 1 Division Health Sciences Education, Faculty of Health Sciences, University of the Free State, Bloemfontein, Republic of South Africa; 2 School of Nursing and Midwifery, College of Health Sciences, University of Ghana, Accra, Ghana; 3 School of Nursing, Faculty of Health Sciences, University of the Free State, Bloemfontein, Republic of South Africa; University of Pretoria, SOUTH AFRICA

## Abstract

**Background:**

Nursing education comprises theory and practice as two complementary parts. Nursing is a practice-based profession, and students are expected to be practically competent in rendering quality care to the deserving population. Clinical training allows nursing students to gain the practical skills required in the nursing profession. In addition to the practical skills, clinical education helps students to learn skills such as problem-solving, decision-making and critical thinking. However, evidence exists that students cannot acquire clinical competence due to multiple factors. These factors are related to students, the clinical environment, and the nurse/faculty. There is a need to identify strategies to enhance clinical teaching and learning in undergraduate nursing education. Therefore, this scoping review aims to identify evidence-based strategies to support clinical teaching and learning in undergraduate nursing education.

**Methods:**

A scoping review method underpinned by Peters et al. (2020) framework was employed. A search string developed from the title of the review was used to search online databases to identify peer-reviewed articles published between January 2011 and September 2023.

**Results:**

Forty studies were included in the review, most originating from the United States. Seven themes were identified: scaffolding the curriculum, transformative teaching and learning approaches, integrating simulation-based education, dedicated education units for clinical placement, applying technology in clinical education, developing interprofessional teamwork and collaboration, and integrating inter-country clinical experience.

**Conclusion:**

The current scoping review has unveiled strategies relevant to nursing students’ clinical teaching and learning. These strategies could guide curriculum developers, programme organisers, nursing teachers, nursing students, and clinical instructors at clinical learning sites in restructuring clinical teaching and learning in undergraduate nursing education to improve clinical education outcomes.

## Introduction

Nursing students do not spontaneously know how to nurse; they must learn nursing. Properly planned and executed clinical teaching and learning within a supportive clinical learning environment (CLE) support nursing students in developing competence in real-life situations [1]. Clinical competence is the ability to solve complex problems employing a combination of knowledge, attitude, and practical skills to meet the nursing/health needs of the populace [2]. The CLE is a key ingredient in competence development for nursing students – inasmuch as it is a complex, multifaceted, and continually changing setting [3]. Nordquist et al. argue that the CLE has a direct influence on nursing student learning, patient outcomes, the well-being of healthcare workers, and the socialisation of all healthcare students to their profession [4].

The physical space, psychological and interaction factors, organisational culture, and teaching and learning components are the four attributes of the CLE with an influence on clinical teaching and learning [5]. The **physical space** reflects the sufficiency of resources that affect clinical teaching and learning, such as equipment, facilities, learning tools and standardised procedures [6,7]. The **psychosocial and interaction factors** of the CLE encompass communication, behaviours and attitudes displayed by qualified healthcare workers, clinical facilitators, and students that influence teaching and learning in the clinical space [8]. The **organisational culture** is related to the healthcare managers’ perception of education, organisational policies related to students’ scope of practice and the provision of quality care to patients [8,9]. Healthcare managers are responsible for guiding and offering adequate human resources to support nursing students [10]. The **teaching and learning components** involve the process and effectiveness of teaching, supervising, and assessing students in the CLE by their clinical facilitators. A blend of the four attributes of the CLE influences the development of competence among nursing students and should be considered critically in the planning and execution of clinical teaching and learning.

More than 4.5 million nurses will be required to augment the healthcare system globally by 2030 [11]. It is questionable whether nursing education institutions (NEIs) can contribute to these high numbers of nurses needed. Debates on appropriate strategies to support NEIs in influencing the numbers of nurses eventually hinge on increasing nursing student intakes, often juxtaposed with limited financial and human resources. In addition, the quality of clinical teaching and learning for undergraduate nursing students is compromised based on reports on the protracted growth of clinical learning opportunities where patients have short hospital stays, increased student numbers, patients opting not to be used for clinical learning, and the unavailability of experienced clinical facilitators [12,13]. Solutions towards increasing the global stock of nurses must be structured to enhance the quality of clinical teaching and learning of undergraduate nursing students.

Rajeswaran [14] reports on the failure of nursing students to integrate their learnt theory into practice in clinical settings in Botswana due to inadequate supervision in the clinical setting, leading to low performance in clinical practices [14]. Other authors identified limited opportunities for hands-on practice in training hospitals, leaving nursing students as mere observers of care [12,13]. In addition, a shortage of nurse educators and clinical facilitators, and large student numbers in nursing education programmes compromise the clinical teaching and learning in nursing education programmes [12,13]. In Norway, nursing students reported a lack of technical mastery of skills and inadequate clinical supervision and clinical placement opportunities [15]. In situations such as pandemics, nurses are the largest group of healthcare workers who play considerable roles in every phase of disaster management [16]. The literature, however, indicates that nurses are insufficiently trained for this purpose [17].

As clinical teaching and learning are faced with a plethora of multifactorial issues [18–21], efforts to improve the quality of nursing education or increase the number of nurses in practice must foreground strategies that enhance the CLE as a supportive space. Fragmentation of strategies to enhance CLE has been reported, necessitating a need for consolidated efforts towards the development of coherent and empirically testable solutions [22].

The current scoping review of the literature unveils reported strategies to enhance clinical teaching and learning in undergraduate nursing education, contributing to the discourse on support for students by nurse educators, clinical instructors, programme directors, and curriculum planners in fostering optimal clinical and learning.

## Methods and methods

A scoping review method described by Peters et al. [23] was used to map significant concepts, evidence characteristics, and types of available evidence related to strategies used to enhance clinical teaching and learning in undergraduate nursing education. This review was conceptualised, implemented and reported according to the guidelines of the Preferred Reporting Item for Systematic reviews and Meta-Analyses extension for Scoping Reviews (PRISMA-ScR) by Tricco et al. [24] and Aromatitis and Munn [25]. The protocol for this current review was developed and registered at Open Science Framework (https://osf.io/5thbj) before the review was done.

The review was conducted using the following steps: developing the review question and search strategy, the screening and selection, the data charting and data analysis, and the presentation of results.

### Developing a review question

The recommended format of population, concept, and context (PCC) guided the construction of a clear and meaningful title and inclusion criteria for the scoping review. The review question was:

*What is known about strategies that enhance clinical teaching and learning in undergraduate nursing education?*

### Search strategy

The search strategy included three components: the search string, the information sources, and the inclusion and exclusion criteria.

#### Search string.

In consultation with other authors and the information specialist, and secondary to several trial searches, two final search strings were developed to conduct two database searches. The first search string was:

(nurs* n3 (Undergraduat* or BSC or Pre-licens* or baccalaureate* or Degree* or bachelor*))

and

(Clinical n3 (Teach* or Learn* or educat*))

and

(“developing countr*” or “developing nation*” or “Low- and middle-income*” or “low and middle income*” or “low-middle income*” or lmic* OR “low resource*” or low-resource* or “low- to middle-income*” or “low income*” or LOW-income*” or MIDDLE-income* or “middle income*” or under-resource*” or “underdeveloped countr*” or “african countr*” or “limited resource*”)

A second search was conducted on the same databases using the following search string:

(nurs* n3 (pre-regist* or preregis* or Undergraduat* or BSC or prelicens* or Pre-licens* or baccalaureate* or Degree* or bachelor*))

and

(Clinical n3 (Teach* or Learn* or educat*)) n5 (advanc* or enhanc*)

and

(Strategy or strategies OR Plan or plans or planning OR Approach* OR Technique* or framework* or guideline*).

#### Information source.

**Fig 1:**
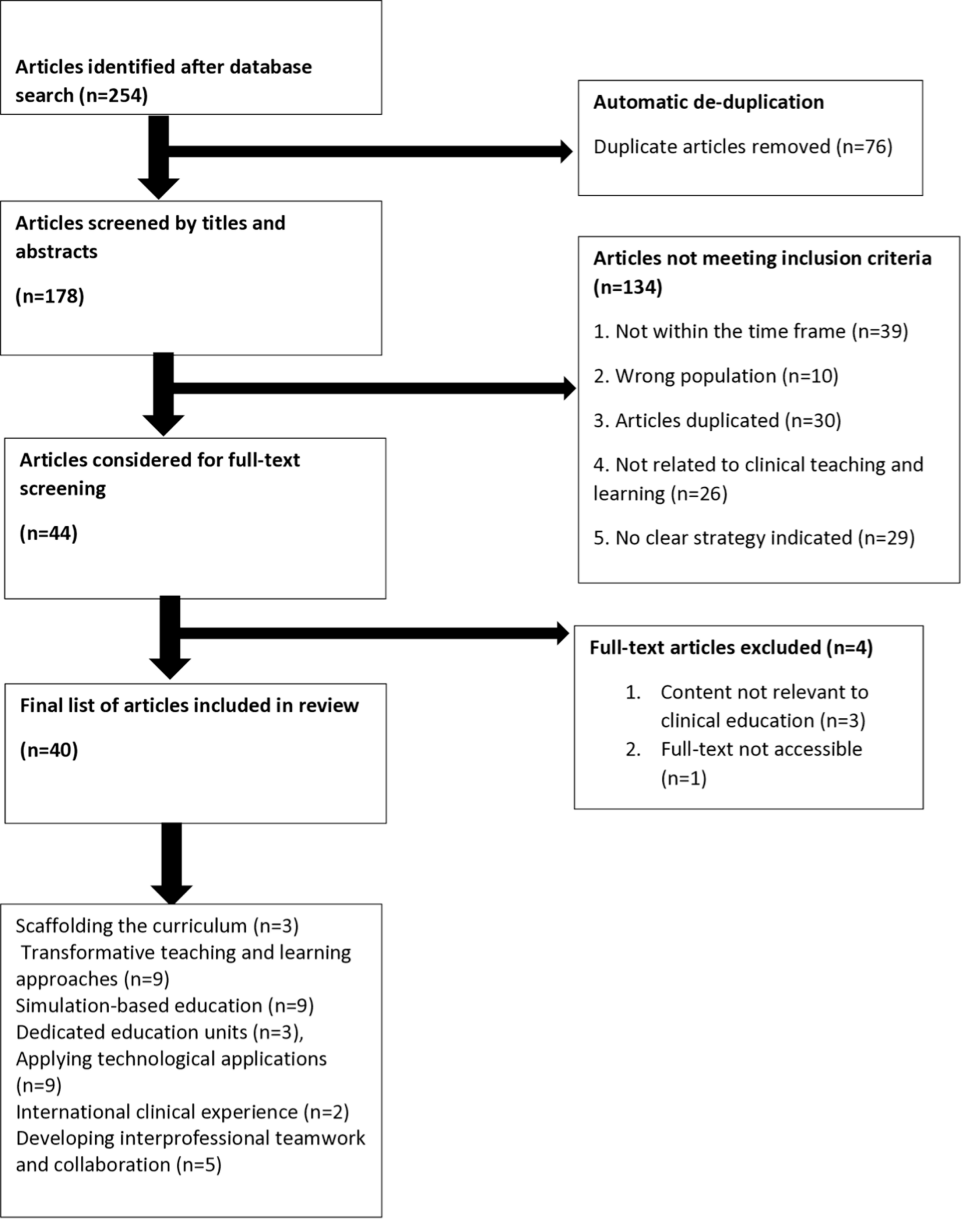
PRISMA-ScR flow diagram.

The electronic bibliographic databases searched to identify research articles relevant to the review question informed by the search string were EBSCOHost databases: Academic Search Ultimate, Africa-Wide Information, Applied Science & Technology Source Ultimate, CAB Abstracts, CINAHL with Full Text, Communication & Mass Media Complete, ERIC, Health Source: Nursing/Academic Edition, Humanities Source Ultimate, MEDLINE, Sociology Source Ultimate. The search was done in three stages. In the **initial search**, the researchers screened the abstract and titles of the identified articles against the review’s eligibility criteria. Subsequently, a librarian from the University of the Free State (UFS) searched for the full text of the articles identified in the initial search. The researchers then screened these full-text articles against the review question and the same eligibility criteria (Fig 1) [26]. In the **final search**, the authors reviewed the reference lists from the three sources:

- all the identified studies from the initial search;
- studies included in the full-text review; or
- studies included in the review.

The titles of the articles were analysed and assessed to ascertain their alignment with the review eligibility criteria.

#### Inclusion and exclusion criteria.

This review included literature on strategies that enhance clinical teaching and learning in undergraduate nursing education. Peer-reviewed journal articles and dissertations published in English between January 2011 and September 2023 were included. Additionally, the review included studies that employed either quantitative, qualitative, or mixed methods designs. Articles that focused on undergraduate nursing students coupled with registered nurses and nursing instructors were included, but those on teaching and learning strategies in postgraduate nursing students and other health profession programmes were excluded.

### Screening and selection

Rayyan, a review management tool [27], assisted the authors in determining and tracking the abstracts and full-text reviews. The initial search yielded 254 reports, and after automatic system de-duplication, 178 reports for the first round of screening remained and were exported to Rayyan (S1 Table). The authors independently screened and selected the evidence. Meetings were held to discuss the individual screening findings and resolve incongruities through discussions. A preliminary examination of the titles and abstracts eliminated 134 articles that did not meet the inclusion criteria. The librarian considered 44 full-text articles, which were screened against the same inclusion criteria. Four full-text articles were eliminated, and 40 full-text articles were included in the final synthesis (Fig 1).

### Data charting

The authors jointly developed a Google form to document the extracted data, and independently piloted this form on three included articles. The form comprised sections that described the characteristics of the studies, including authors and year of publication, country, title of the article, design, purpose of study, sample size, population, name of strategy, main findings, and recommendations (S2 Table). Any adjustment or modification of the form was duly documented to affirm the iterative nature of the data extraction process [23].

### Data analysis

The analysis of the extracted data entailed tallying the frequencies of the characteristics and inductive analysis. Regarding the characteristics of the included articles, KAH extracted the data and tallied the frequencies of the year of publication, country, title of the article, design, purpose of study, sample size, population, and name of strategy. Inductive thematic analysis was done, specific to the population and other extracted data. KAH performed the initial analysis and co-coded with CNN to generate 36 sub-themes which were clustered into seven themes; AOA and FN verified the outcomes of the coding and co-coding processes and the results. (Table 5).

**Table 5:** Identified themes and sub-themes of strategies to enhance clinical education of nursing students.

Strategies to enhance clinical education of nursing students (themes)	Techniques (sub-themes)	Sources and/or references
Scaffolding the nursing curriculum	Deliberate sequencing of clinical learning experiences in the curriculum	Neilson et al. [28]
	Integrating the usage of smartphones into the curriculum	Day-Black [29]
	Integrating student dyads (collaborative learning) into foundational stages of clinical education	Austria et al. [30]
Transformative teaching and learning approaches	Embedding experiential learning theories in clinical education	Landers et al. [31]; Rodrigueze-Garcia et al. [32]
	Encouraging peer-assisted learning and collaborative learning in clinical education	Carey et al. [33]; Austria et al. [30]; Farzi et al. [34]
	Employing photovoice in clinical education	Gallagher et al. [35]
	Improving clinical preceptor roles	Dias et al. [36]
	Using the nursing process as an educational model	Farzi et al. [34]
	Implementing clinical associate resources and support orientation programme	Hunt et al. [37]
	Using multiple intelligence teaching approach	Sheahan et al. [38]
	Iterative problem-solving approach	Liu et al. [39]
	Conducting post-clinical conference	Lee and Lapum [40]
Integrating simulation-based education	Simulation as a pedagogic method	Bϕ et al. [41]; Farzi et al. [34]
	Simulation-based programme	Liaw et al. [42]
	Use of high-fidelity simulation	Bates et al. [43]; Coffey et al. [44]; Roop [45]
	Clinical pause approach in simulation debriefing	Kacalek et al. [46]
	Engaging in virtual simulation	Panepucci et al. [47]
	Adopting train-the-trainer model	Coleman et al. [48]
	Interprofessional mass casualty incident simulation	Strout et al. [49]
Dedicated education units for clinical placement	Creating dedicated education units at clinical sites	Rhodes et al. [50]; Dorcy et al. [51]; Smyer et al. [52]
Applying technology in clinical education	Utilisation of smartphones in clinical education	Day-Black [29]
	Adopting instant messaging	Pimmer et al. [53]
	Collaborative clinical learning mobile application	Lee et al. [54]
	Introduction of desktop-based virtual reality platform	Botha et al. [55]
	Mobile augmented reality technologies	Garrette and Jackson [56]
	Virtual patient-based social learning	Hwang et al. [57]
	Collaborative learning in a virtual team	Thomson et al. [58]
	Development of digital educational resources using design thinking framework	Laugaland et al. [59]
	Online problem-based learning	Adongo et al. [60]
Developing interprofessional teamwork and collaboration	Peer-support approach	Cheraghi et al. [61]
	Bearing witness approach	Minden [62]
	Clinical-partnership model	Tang and Chang [63]
	Improving faculty–practice relationship	Farzi et al. [34]
	Interprofessional collaboration Interprofessional mentoring	Waston [64] Lait et al. [65]
Integrative inter-country clinical experience	International clinical-placement programme	Ulvund and Mordal [66]; Sundal and Ulvund [67]

## Results

The identified characteristics and the strategies are presented below.

### Characteristics

The general characteristics of the 40 full-text articles are described below.

In this scoping review, 40 full-text articles were considered. The articles comprised a range of studies over a 13-year period and were from:

- the United States (n = 16);
- Canada (n = 3);
- two each from Ireland, Iran, and Ethiopia (n = 6); and
- one study each from Australia, China, Hong Kong, New Zealand, Nigeria, Norway, Pakistan, Singapore, South Africa, South Korea, Spain, Taiwan, Tanzania and Madagascar, Uganda, and the United Kingdom (n = 15) (Table 1).

**Table 1:** Countries where studies were conducted (n = 40).

Characteristic	Number of studies (%)
**Country**	
**United States**	16(40.00)
**Canada**	3 (7.50)
**Ireland**	2 (5.00)
**Iran**	2 (5.00)
**Ethiopia**	2 (5.00)
United Kingdom	1 (2.50)
**China**	1 (2.50)
**Australia**	1 (2.50)
**Norway**	1 (2.50)
**New Zealand**	1 (2.50)
**Pakistan**	1 (2.50)
**Spain**	1 (2.50)
**South Korea**	1 (2.50)
**Nigeria**	1 (2.50)
**Uganda**	1 (2.50)
**Hong Kong**	1 (2.50)
**Tanzania and Madagascar**	1 (2.50)
**Singapore**	1 (2.50)
**Taiwan**	1 (2.50)
**South Africa**	1 (2.50)

The 40 articles included were published in different years as follows: 2022 (n = 8), 2015 (n = 6), 2018 (n = 5), four articles each in 2013 and 2021 (n = 8), three articles each in 2017 and 2019 (n = 6), two articles each in 2011, 2012 (n = 4) and one article each in 2016, 2020 and 2023 (n = 3) (Table 2).

**Table 2:** Year of publication (n = 40).

Year of publication	Number of studies (%)
**2011**	2 (5.00)
**2012**	2 (5.00)
**2013**	4 (10.00)
**2015**	6 (15.00)
**2016**	1 (2.50)
**2017**	3 (7.50)
**2018**	5 (12.50)
**2019**	3 (7.50)
**2020**	1 (2.50)
**2021**	4 (10.00)
**2022**	8 (20.00)
**2023**	1 (2.50)

Different designs were employed by the studies included in this review, namely qualitative (n = 13), quantitative (n = 8), mixed method (n = 5), and non-specified (n = 14) (Table 3).

**Table 3:** Study designs (n = 40).

Study design	Number of studies (%)
**Qualitative**	13 (32.50)
**Quantitative**	8 (20.00)
**Mixed method**	5 (12.50)
**Non-specified**	14 (35.00)

The population of the studies comprised nursing students (n = 25), nursing students and instructors (n = 8), nursing instructors (n = 3), registered nurses (n = 2), and population not reported (n = 2) (Table 4).

**Table 4:** Population (n = 40).

Population	Number of studies (%)
**Nursing students**	25 (62.50)
**Nursing students and instructors**	8 (20.00)
**Nursing Instructors**	3 (7.50)
**Registered Nurses**	2 (5.00)
**Not reported**	2 (5.00)

### Strategies to enhance clinical education for nursing students

After thematic analysis, seven themes and 36 sub-themes were identified. These are reflected in Table 5.

### Scaffolding the nursing curriculum (Theme 1)

Some of the articles proposed a review of the nursing curriculum as an approach to enhance clinical education in undergraduate nursing. Scaffolding the nursing curriculum was therefore identified as a strategy after clustering three sub-themes.

The first sub-theme was labelled ‘Deliberate sequencing of learning experiences in the curriculum’*.* Nielson et al. [28] argue that clinical learning experiences should be incorporated sequentially into the nursing curriculum in this order: skill-based, case-based, concept-based interventions, focused direct client care, and integrative experiences.

‘Integrating the use of smartphones into the curriculum’ was the second sub-theme.

Day-Black [29] proposes integrating smartphones into the nursing curriculum as a tool to support teaching and learning. The author, however, reports a slow adoption of the innovation in the implementation setting, citing the perception by the nursing teaching staff of smartphones as a distraction in the classroom rather than a potential learning tool. Day-Black, therefore, suggests that evidenced-based research is required to investigate and support smartphone integration into nursing programmes [29].

The third sub-theme was ‘Integrating student dyads into the foundational stages of the curriculum’. In the study by Austria et al. [30], students suggested that peer dyads are most appropriate in the early or foundational stages of the nursing programme amidst the availability of a clinical instructor to provide instruction and support to novice students [30].

### Transformative teaching and learning approaches (Theme 2)

This theme describes the varied strategies to enhance clinical teaching and learning in undergraduate nursing education. This theme was derived from clustering nine sub-themes.

**‘**Embedding experiential learning theories in clinical education’ was the first sub-theme.

Two articles identified experiential learning as a key strategy to enhance clinical teaching and learning in undergraduate nursing [31,32]. Landers et al. [31] propose Steinaker and Bell’s taxonomy, which is grounded in experiential learning theory and aligned with the constructivist origins of teaching and learning for clinical learning. Reportedly, this taxonomy incorporates teaching strategies for planning, sequencing, implementing and evaluating the experience of teaching and learning. The authors documented that the model has five sequential levels: exposure, participation, identification, internalisation, and dissemination [31]. Landers et al. further state that the first four levels in the sequence are appropriate for undergraduate students, while the fifth level is apposite for the graduate nurse.

Again, the report intimated that the five levels are inherently connected but largely dependent on the preceptor or mentor to inspire the learner to partake in the learning experience. During the exposure stage, the learner is first exposed to the learning experience, and the preceptor or mentor uses audio or visual materials, engages in questioning, and performs activities to arouse the student’s interest [31].

In the second level of participation, the learner is physically and mentally active in the learning process. During this stage, the preceptor or mentor provides supportive feedback to the learner or intercedes if the learner encounters an obstacle or communicates learning successes made by the learner. The identification stage stems from the learner’s emotional attachment and intellectual absorption of the learning experience, during which the learner becomes acquainted with the new knowledge and acquired skills. At this level, learners are reported to have become self-directed in the learning process by seeking additional learning opportunities and resources. As the learner graduates to the internalisation level, the preceptor or the mentor moderates the learning process but sustains the learner’s engagement, finds increasingly difficult preps, and engages in more unconventional questioning regarding new learning [31]. The taxonomy levels guide the preceptor or mentor in measuring the progress of the nursing student through each clinical placement and at the end of each year of the programme [31].

Rodrigueze-Garcia et al. [32] indicate that preceptors rely on their experience to facilitate the development of the technical skills of students, and students are offered the opportunity to experiment with procedures and learn from their mistakes. Preceptors refer to students’ mistakes, give the correct option, and ask students to ponder over the correct option to gain experiential knowledge. Students are given professional roles to help them integrate knowledge acquired from preceptors [32].

The second sub-theme was *‘*Encouraging peer-assisted learning and collaborative learning among nursing students. Peer-assisted learning [33,34] and collaborative learning [30] were identified as strategies in support of transformative teaching and learning approaches. Peers were reported to have assumed the role of and acted as informal facilitators to their peers. They also offered guidance and advice to their colleagues, which helped with easy information assimilation. Through peer-assisted learning, students were reported to have demonstrated learning and development in interactions during clinical practice and planning of patient care. It was further documented that peer-assisted learning created the platform for networking, shared learning, and navigation within the clinical environment. Similarly, Austria et al. [30] report that collaborative learning among students increased confidence in students and staff.

Patients mentioned that they received quality nursing care and that their satisfaction increased. It was further noted that collaborative learning among nursing students made cognitive processing and decision-making a shared responsibility. Again, this approach resulted in efficient completion of clinical tasks, served as a support base for students, and decreased anxiety among students [30]. Contrarywise, the collaborative learning approach led to a longer time for completion of a task, missed learning opportunities among students as a result of the negotiation of a task, and conflict among student dyads as a result of the dominance of one student performing most tasks [30].

The current review identified photovoice as one of the transformative teaching and learning approaches that can be employed. Gallagher et al. [35] found that students used photographs as reference points during their critical dialogue. The photographs made it possible for students to have an entire view of a concept. The authors suggest that the photovoice medium could be used to provide students with an enjoyable activity that would lead to reflection and critical thinking about their practices in nursing [35].

The fourth sub-theme was labelled ‘Improving clinical preceptor roles’.

The current scoping review highlights the roles of clinical preceptors from the viewpoint of students, teaching staff and administrators [36]. Students indicated that clinical preceptors’ knowledge and experience helped them discharge their preceptor roles. Students further reported that clinical preceptors employed teaching methods, such as demonstrations and return demonstrations in teaching. The non-threatening attitude of clinical preceptors reduced the anxiety levels of students. Additionally, students revealed that involvement of clinical preceptors in their clinical education enhanced their self-confidence in performing nursing procedures. According to the students, clinical preceptors discussed new learning opportunities with them and played leading roles during post-clinical conference discussions. Students also stated that clinical preceptors’ long-standing and good working relationship with other staff created a platform for them to collaborate easily with other healthcare team members [36].

Teaching staff reported that clinical preceptors were receptive to taking assignments and initiatives, sought guidance whenever necessary, and accepted delegated duties. They further indicated that clinical preceptors were competent in clinical skills, mainly in the areas where they worked as professionals [36]. Nursing administration was reported to have viewed the hiring of clinical preceptors by nursing schools as a laudable initiative. Additionally, hiring clinical preceptors complies with accreditation requirements from regulatory bodies [36]. Also, nursing administration indicated that hiring clinical preceptors was a cost-saving measure as the latter were hired for only one semester without any additional benefits [36].

Aside from the documented roles of clinical preceptors, students, teaching staff, and administrative staff reported some challenges. For instance, students and teaching staff reported that clinical preceptors had difficulty integrating theory into practice and in providing timely feedback. It was further reported that teaching staff mentioned difficulty in maintaining students’ anecdotal records, handling difficult students, a lack of experience and planned mentorship. For nursing administration, the challenge reported was the recruitment of new clinical preceptors every semester [36].

‘Using the nursing process as an educational model’ was the fifth sub-theme. This sub-theme was reported in one article included in this scoping review. Farzi et al. report that using the above model helped students to develop their critical thinking skills [34].

The sixth sub-theme was ‘Implementing the clinical associate resources and support (CARS) orientation programme’. One article included in this review reported the need to execute a clinical associate resource and support orientation programme [37] to ensure transformative teaching and learning approaches are achieved. The article reported that clinical associates were trained in effective teaching strategies, pre- and post-clinical conferences, critical thinking activities, time management, teaching staff–clinical associate mentoring relationships, and communication channels. Other reported focal areas of the training included student evaluation, recommendation and remediation, care planning, student policies and procedures, and difficult student behaviours [37].

The seventh sub-theme was labelled ‘Multiple intelligence teaching approach’ (MITA). In the current review, the MITA was reported as one of the ways to implement transformative practices to enhance clinical teaching and learning in undergraduate nursing education [38]. The authors conducted a randomised controlled trial (RCT) to assess whether the MITA is an effective method of teaching clinical skills to first-year undergraduate nursing students. They reported sensing as the preferred learning style of students, while intuition was preferred least. Interpersonal intelligence was the highest type of intelligence, and naturalistic intelligence, the weakest. The MITA was reported as a diverse learning method that supports the learning of clinical skills. The use of music during the use of the MITA helped to reduce stress [38].

The ‘Iterative problem-solving approach’ was the eighth sub-theme. The study by Lui et al. [39] was intended to reduce or eliminate the identified stressors and enhance nursing students’ clinical experience by implementing an iterative problem-solving approach. The authors documented everyday stressors and their corresponding initial and refined solutions among four cohorts of nursing students. Students were reported to be stressed because of a lack of confidence in the clinical placement site. As an initial solution to this stressor, there was an increase in the compulsory practice in the school laboratory. This initial solution was refined to inviting teaching staff or senior students to supervise students’ practical skill training in the school laboratory and assigning each first-year student to senior nursing students as mentors.

Another stressor among nursing students mentioned was the fear of making mistakes at the clinical placement site. The initial solution proposed was to increase the compulsory practice hours of students in the school laboratory. As a revised solution, the number of students under a preceptor was reduced, and an opportunity was created for high-performing senior students to share their knowledge and experience. The final solution for the fear of making mistakes was conducting a workshop for students at the beginning of the academic year on peculiar topics such as stress management, patient safety, and infection prevention practices.

Besides, students were reported to have been stressed due to workload and overwhelmed by responsibility. The foremost solution was to reduce students’ assignments. Additional solutions were integrating assignments from preceptors and teaching staff of the school, engaging students in team-based learning, and discussing with nurses at the clinical placement sites not to consider students as additional workforce or give them responsibilities above their competency level.

The final stressor emanated from the disconnection between what was learned and the actual clinical practice. The preliminary solution was to conduct pre-and post-clinical briefings with preceptors every semester after students’ clinical placement, while the final solution reported was organising a clinical preceptor training programme to improve precepting skills [39].

‘Conducting post-clinical conferences’ was the ninth sub-theme.

Post-clinical conference was reported as a transformative teaching and learning approach [40]. This study reported that the active participation of students in post-clinical conferences created a platform for reflective practice and an avenue to discuss students’ experiences in blending clinical experience with theoretical knowledge. Students were reported to have learned from each other, and identified a common interest among students during the post-clinical conference [40].

### Integrating simulation-based education (Theme 3)

This theme describes simulation as a strategy to enhance clinical teaching and learning in undergraduate nursing education. The theme comprised seven sub-themes.

The first sub-theme was identified as ‘Simulation as a pedagogical method’.

In the study of Farzi et al. [34], it was reported that nursing students and clinical educators suggested simulation as a strategy to enhance clinical education. In their mixed method study, Bo et al. [41] provided a quantitative report indicating that students rated scores of 4 or above on a 5-point Likert-type scale on educational practices, such as active learning, collaboration, diverse ways of learning, and high expectations. Their report indicated that simulation built the competence and confidence of the students, and supported their active learning. In addition, the authors found that playing active roles in a simulation session and a lack of prior knowledge of the simulation scenarios caused a heightened level of anxiety among students. Besides, students were reported to be expectant of debriefing after simulation sessions [41].

‘Simulation-based programme’ was the second sub-theme. Liaw et al. [42] explored nursing students’ experiences on how a simulation programme prepared them to transfer their clinical performance to encounters with deteriorating patients in wards. They reported factors influencing their ability to transfer their learning from the simulation laboratory to a real patient care setting were:

- retrieval of knowledge from memory;
- retention of learning in the long-term memory; and
- using mnemonics as transfer tools; recognising similar situations; but also –
- a sense of familiarity between actual clinical experience and prior simulation experiences and
- feeling calm as an emotional response to a deteriorating patient situation.

A feeling of stress was stated as a barrier to transferring simulation learning. Additionally, the study found that strategies that could enhance the existing simulation education programme to facilitate the transfer of learning included:

- realism by replacing manikins with simulated patients;
- variations in simulation scenarios;
- self-directed learning;
- revisions; and
- multimedia resources, such as videos [42].

The third sub-theme was ‘Using high-fidelity simulation’. Three of the studies used high-fidelity simulation (HFS) as a strategy in clinical education [43–45]. Bates et al. [43] compared the difference between an observer role and an active nursing role in terms of student anxiety and learning outcomes in HFS. Students who played the active nursing role in the HFS had increased pre-simulation anxiety and decreased post-simulation anxiety. It was further reported that students had an improved ability to provide patient care, collaborate with members of the healthcare team and peers, and engage in problem-solving skills. Besides, it was documented that HFS effectively improved students’ confidence in practice.

The report by Coffey et al. [44] showed that of the participating students –

- 98.9% strongly agreed that HFS enhanced their learning;
- 97.2% said it developed their clinical reasoning skills;
- 96.9% reported developing their clinical decision-making ability; and
- the majority (99.3%) indicated that the HFS was a valuable experience [44].

Roop [45] compared and assessed the traditional clinical experience with HFS and evaluated its effectiveness as a replacement for the traditional clinical experience. Roop concluded that HFS met the learning needs of the nursing students better than the other two clinical learning environments. The screen-based simulation (virtual-simulation) environment was further reported to have met the students’ learning needs on the critical thinking subscale better than the other two clinical environments. Besides, the traditional clinical environment met the students’ learning needs in the teaching–learning dyad subscale better than the two clinical learning environments. In addition, the high-fidelity face-to-face simulated clinical environment met the students’ learning needs in the holism subscale better than the two other clinical learning environments. Furthermore, HFS was documented to have utilised fewer nursing educators than educating the same number of students required for clinical hours in the ward [45]. Conversely, inadequate time for pre-briefing was reported as a shortcoming associated with the high-fidelity face-to-face simulation. Roop consequently recommends an improvement in the pre-briefing phase and offering time for nursing skills in the laboratory to enhance students’ learning experience during high-fidelity simulation [45].

‘Implementing a clinical pause approach in simulation debriefing’ was named the fourth sub-theme, as reported by Kacalek et al. [46]. They found that the clinical pause facilitated timely knowledge construction, contextual thinking, and clinical judgement of students. Furthermore, the teaching staff perceived that the clinical pause approach provided timely feedback and reflection-in-action and enhanced students’ thinking skills. Moreover, the timing of the clinical pause promoted rich discussion and robust learning based on recent recall instead of remote recollection [46].

The next sub-theme was ‘Engaging virtual simulation’. Panepucci et al. [47] state that students agreed that simulation was effective in pre-briefing, learning, confidence, and debriefing. Students also viewed the virtual simulation as an interactive way to enhance learning. Students found that stopping points allowed them to engage with educators. These stopping points during the virtual simulation allowed students to project their thoughts and opinions, practice prioritisation skills, and make decisions before the simulation evolved. The authors additionally report that the students were able to determine appropriate assessments and interventions the nurse should implement, and they observed the results of the nurse’s actions through coaching during each pause. The report further showed that students observed and enjoyed professional enacted roles. Teaching staff and simulation educators provided immediate feedback to students on their decision-making regarding the virtual simulation [47].

Another sub-theme was ‘Adopting the train-the-trainer model’. Coleman et al. [48] conducted a regional simulation survey to describe an academic partnership between nursing programmes and service partners that used a train-the-trainer model and other activities to promote simulation. The report noted an increase in the purchase of manikins and an increased development of simulation laboratories. A key challenge was the need for adequately educated and trained laboratory administrators and technical support personnel [48].

The final sub-theme was labelled ‘Interprofessional mass casualty incident simulation’.

Strout et al. [49] report on an intervention to prepare pre-licensed nursing students to respond effectively to disasters. Most participating students (96%) indicated that the simulation increased their confidence in responding to a disaster. All participants’ overall event rating (n = 40) was 94.25% [49].

### Dedicated education units for clinical placements (Theme 4)

A dedicated education unit (DEU) for clinical placement was identified as a main strategy to enhance clinical teaching and learning in undergraduate nursing education. Creating DEUs at clinical learning sites was reported by three studies [50–52] and was reported as a key way of enhancing clinical teaching and learning in undergraduate nursing education. Rhodes et al. [50] indicated the benefits and degree of satisfaction that educating student nurses in a DEU provides to the students, DEU nurses, and teaching staff.

Firstly, students were reported to be satisfied with the learning environment at the DEU, had an excellent relationship with the staff, and felt like part of the healthcare team. Students were treated with respect, had a one-on-one engagement with nurses and the opportunity to ask questions, with 90% being answered satisfactorily. Students were also encouraged to practise independent problem-solving skills at the DEU.

Secondly, it was reported that DEU nurses were satisfied with their involvement with nursing students, and working at the DEU helped the nurses to embrace team building and leadership, and encouraged them to seek further education. Additionally, DEU nurses received support from teaching staff regarding how to manage students.

Thirdly, it was reported that the involvement of DEU nurses lightened the clinical instructor role of the teaching staff. Other benefits of DEU include fostering academic–practice partnerships and critical thinking [50].

Students who have experienced the DEU are highly employable and successful in state examinations, as noted by Dorcy et al. [51]. In addition, the job satisfaction of preceptors at the DEU increased [51]. Smyer et al. [52] reported no significant differences between participants in DEU and traditional clinical settings [52]. Rhodes et al. [50] cite some challenges of the DEU from the perspectives of students and DEU nurses. On the one hand, some students indicated that the DEU nurses were not trained, ready, or committed to meeting their responsibilities to students; students had different time schedules with their DEU nurses; and there was overcrowding at the DEU at certain times. On the other hand, DEU nurses indicated that managing students in terms of patient care was a challenge; there was poor assignment of students to the DEU, and recognition of DEU nurses was limited to only those in charge of students [50].

### Applying technology in clinical education (Theme 5)

This theme describes the application of technology in clinical education as a strategy to enhance undergraduate nursing. This strategy was derived from clustering nine sub-themes.

‘Utilisation of smartphones in clinical education’ was captured as the first sub-theme.

Day-Black [29] specified that utilising smartphones in clinical education helped students to download useful applications, such as drug references, medical information, drug calculation or dosing, and electronic versions of textbooks that can be carried by the student in one device that can fit into a pocket to support clinical learning activities. Additionally, it was intimated that smartphones helped to promote patient safety through an informatics-based approach to documentation during clinical encounters, retrieved patient safety-related information at the point of care, and developed procedural skills [29].

The second sub-theme was ‘Adopting instant messaging’*.* In one study, Pimmer et al. [53] investigated the use of WhatsApp and its correlation with some socio-professional indicators. Nursing students’ use of WhatsApp during their clinical placements was associated with all the socio-professional factors, but most strongly with the social capital students maintain through their connections with friends and fellow students. Furthermore, positive correlations were identified between nursing students’ use of WhatsApp during placement, their professional identity, and their placement satisfaction. Contradictorily, negative associations were reported between WhatsApp use during placement and the students’ feelings of professional isolation [53].

Determinants of the WhatsApp ease of use include attitude, usefulness, and subjective norms. The perceived usefulness of WhatsApp as a tool to enhance communication between students and nurses and its ease of use were associated with use during clinical placements and in general [53]. Nonetheless, subjective norms and attitudes were significantly correlated only with WhatsApp use in general but not with WhatsApp use during clinical placement. A positive correlation between perceived usefulness and social capital was also reported [53].

**‘**Collaborative clinical learning with mobile application’ was labelled as the third sub-theme.

In their study, Lee et al. [54] developed a mobile application for use in a paediatric clinical environment to enhance student activity and self-regulated learning and to facilitate collaboration between the preceptor and the nursing teaching staff. The authors explored experiences of the use of the application. They found that using the mobile application by students facilitated collaborative learning through real-time interactions and communication. Preceptors and students were documented to have had increased opportunities to interact through the mobile application. It was further stated that students received prompt feedback from preceptors, as they shared the same materials provided in the mobile application. Again, it was found that using the mobile application enabled students to engage in active learning. The report further indicated that students had access to video clips and other educational resources, which helped them practice nursing skills or acquire nursing knowledge anytime and anywhere. Furthermore, it was stated that the mobile application enhanced students’ critical thinking abilities, and students were cited to have executed the nursing process dynamically and on time. Students were also stated to have completed the nursing process promptly during clinical practice and demonstrated the ability to critically prioritise patient problems due to the limited space allocated to the nursing process on the application. Other remarks documented were that using the mobile application helped manage work efficiently and saved time and paper [54]. Conversely, challenges reported to have hindered the adoption of the mobile application technology included data loss through technical errors, charging issues, and unstable network connectivity [54].

Additionally, *‘*Introduction of a desktop-based virtual reality platform’ was identified as another sub-theme. Botha et al. [55] conducted a feasibility assessment on a desktop-based virtual reality platform developed to measure students’ learning experiences during COVID-19, which restricted work-integrated learning. The authors remarked that the desktop reality platform afforded students in quarantine or isolation an opportunity for the continuous learning process. Furthermore, the virtual clinical learning platform could be re-used and re-engaged at students’ convenience. Additionally, it was documented that the students could develop clinical reasoning related to clinical learning. It was, however, remarked that students would not be able to develop psychomotor skills via this platform. System-related issues in terms of connectivity and technical support must therefore be addressed [55].

The fifth sub-theme was ‘Mobile augmented reality technologies’*.* Garrette et al. [56] report on a pilot study that was designed to explore a new mobile augmented reality technology, which has the potential to enhance the learning of clinical skills from the perspectives of nursing students and instructors in the clinical skills laboratory. On the one hand, most students indicated that the learning resources reflected high technical quality, and the difficulty levels were appropriate and focused on specific skills [56]. Students found the learning resources to be accessible anywhere and organised in a consistently logical fashion for ease of use. Besides, they preferred internally created videos since the external videos were found to be confusing. Even though students were reported to have preferred video resources, they also wanted videos to be used as supplemental material and not that it should replace the practical demonstration sessions in the skills laboratory. On the other hand, the majority of the instructors agreed that the augmented reality resources could help students learn skills. Similar to the views of students, instructors preferred video-based resources [56].

The authors named scanning and downloading difficulties, internet speed and connectivity issues, small phone screens, a lack of access to smartphones, and instability of applications as challenges associated with the use of augmented reality technologies. They indicated that students and instructors required more training to be able to use augmented reality [56].

**‘**Virtual patient-based social learning’ was the sixth sub-theme. One study by Hwang et al. [57], integrated a virtually based interactive system to provide an innovative educational model for nursing students. From different perspectives of educational objectives, the impacts of the system were demonstrated by referring to the social learning theory. Hwang et al.’s findings indicated that the virtual patient-based social learning approach improved students’ learning achievement and self-efficacy in the intervention group more than in the control group when compared to the conventional didactic education approach. In terms of communication skills, the intervention group had significantly higher post-test scores than the control group [57].

The seventh sub-theme was ‘Collaborative learning in a virtual team’.

Thomson et al. [58] explored an innovative solution for creating change in the delivery of undergraduate nursing education. The report [58] indicated that the motivation, deep understanding of nursing pedagogy, experience as a registered nurse, and positivity and teamwork of the academic team were key drivers to enable the timely continuation of nursing students’ learning within the course. This approach enhanced teamwork, demonstration of leadership, and relevant and applicable knowledge, emphasising work readiness as future registered nurses. Furthermore, the approach enhanced clinical skills, namely venepuncture, vaccination, and the unique opportunity for nursing students to gain clinical experiences at managed isolation or quarantine facilities (MIQFs). The multi-disciplinary team established a safe and supportive learning environment for nursing students through procedures and protocols. This approach resulted in the employment of the graduates of the bachelor nursing programme [58].

Another sub-theme was ‘Development of digital educational resources using design thinking framework’. One of the studies, that of Laugaland et al. [59], reported on the seven-phase methodological development process of an interactive digital educational resource to enhance the quality of clinical education in nursing homes.

- In the **empathise** phase, the researcher strives to understand and gain insight into stakeholders’ needs.
- The **define** phase aims to determine specific, meaningful challenges to be addressed in the interactive digital educational resource.
- The third phase, the **ideation** phase, entails a joint workshop with all stakeholders.
- In phase four, **prototyping a digital educational resource** to enhance mentorship practices takes place.
- During phase five, **pilot testing and evaluation** are undertaken to refine and spark new ideas. Feedback is solicited from users about the prototype that has been created.
- Phase six focuses on **refining the prototype and solutions** (design, content, and functionality of the educational resource).
- In phase seven, **pilot testing and evaluation** of the digital educational resource targeting all stakeholder groups is done [59].

The last sub-theme is ‘Online problem-based learning’*.* Adongo et al. [60] explored the perceptions of students regarding the benefits of online problem-based learning (PBL) and its associated effect on the learning experience of students. On the one hand, online PBL allowed continued learning during the lockdown, and was noted to be flexible, and it enhanced self-drive and opportunity for work, solved infrastructure problems, and protected students from COVID-19 infection. On the other hand, students were reported to have perceived that there was less learning online compared to face-to-face sessions because of reduced learner engagement, concentration, motivation and peer-to-peer learning, as well as limited opportunities for practical sessions. Online learning also increased students’ workload in the form of several assessments assumed to reduce learning. Additionally, online tutorials were perceived to reduce the acquisition of soft skills, such as confidence, communication, leadership, and practical or clinical skills [60].

### Developing interprofessional teamwork and collaboration in clinical education (Theme 6)

This theme focuses on communication, building working relationships, and networking among key stakeholders in the clinical teaching and learning environment. Developing interprofessional teamwork and collaboration in clinical education as a main strategy was derived from six sub-themes.

The first sub-theme was the ‘Peer support approach’*.* The study by Cheraghi et al. [61] aimed to determine the effect of a peer support approach on nursing students’ communication skills when interacting with hospitalised children and their parents in the paediatric setting of a large tertiary hospital in Hamadan City, Iran. The authors found that the peer support approach promoted the communication skills of undergraduate nursing students [61].

‘Bearing witness approach’ was identified as the second sub-theme.

In the bearing witness approach, Minden [62] used undergraduate nursing clinical courses to teach multiple intersecting, subtle, and nuanced intra- and interpersonal micro-abilities that underpin therapeutic effectiveness. It was reported that the approach helped students to comprehend mental illness and the people living with it better, and it also increased students’ empathy and confidence to intervene. Again, it was argued that the bearing witness approach made students appreciate that interpersonal competence results from experimentation, error, reflection, and correction rather than luck and inheritance. Observing an experienced interviewer helped participating students learn the components of skilful interviewing, such as empathising, paraphrasing, and validating. Additionally, students found that watching each other mumble, stumble, and fumble during the interviews helped them acquire interview skills [62]. Furthermore, students refined their knowledge and skills in the interviews that they considered essential, which could be applied in any context for a rewarding and productive relationship [62].

**‘**Clinical partnership model’ was named the third sub-theme. Tang and Chang [63] explored students’ clinical learning experiences using the clinical partnership model and found that the model facilitated good collaboration between clinical and school teachers. The authors hinted that other staff were willing to teach the nursing students in the absence of their clinical teachers. Furthermore, the report stated that teachers from the school monitored students at the clinical site, provided feedback, and shared their experiences, which facilitated student learning. Again, the report stated that clinical teachers were experienced, and they shared their expertise with students. Additionally, the familiarity of clinical teachers with the ward enabled students to adapt to the clinical environment, resulting in less tension among students and minimal risk of committing mistakes in the clinical setting. Clinical teachers were also found to be experienced and shared their expertise. Also, they provided more clinical learning opportunities to students than school teachers. Students were exposed to more clinical tasks, role-modelled and learned the professional roles of nurses The authors found that students learned from clinical practice that knowledge is flexible, unlike the fixed knowledge in textbooks. Besides, the clinical partnership model fostered students’ connection with the healthcare team [63].

The fourth sub-theme was ‘Improving faculty–practice relationship’*.* Farzi et al. [34] found that clinical educators viewed the relationship between faculty and practice as an effective factor in the clinical education process. The participants indicated that cooperation without fear, blame, and/or suppression between the faculty and practice would create an atmosphere conducive to allaying the fears and concerns of students and supporting clinical education [34].

Another sub-theme was ‘Interprofessional collaboration’*.* Waston [64] provides evidence on the development of interprofessional competence of nursing students. Students are reported to have learned interprofessional collaboration and teamwork, developed self-confidence, felt valued and respected, and their activities – such as checking vital signs – were recognised by other healthcare team members. Part of Waston’s findings also indicated that, through interprofessional collaboration, students had the opportunity to undertake ward rounds with doctors and developed effective open and confident communicative skills [64].

Additional findings included:

- interprofessional collaboration enhanced professional identity;
- a comprehension of professional roles and responsibilities;
- an experience of interprofessional cohesiveness due to a shared purpose of providing care to the deserving population;
- professional, respectful relationships among team members; and
- autonomy within the students’ professional scope improved their nursing skills [64].

The final sub-theme was ‘Interprofessional mentoring’*.* Lait et al. [65] describe a study that implemented and evaluated interprofessional mentoring between staff and students. The researchers found that interaction between students and other professionals and learning beyond the classroom was enhanced. They found that other healthcare providers assumed the role of mentors and connected students to staff across disciplines. Students had the opportunity to observe how other healthcare providers do their work, which also increased students’ knowledge of the roles of other healthcare providers in implementing interprofessional mentoring. This helped nursing students understand that their roles involve working with others [65].

### Integrative interv-country clinical experience (Theme 7)

This theme describes the relevance of inter-country clinical placement as a strategy to enhance clinical teaching and learning in undergraduate nursing education. Two of the studies included in this review [66,67] used international clinical placement as a strategy in the clinical education of nursing students. The international placement programme found that students developed cultural awareness, competence, awakening, and sensitivity. In addition, the authors found that the students noted the resource limitations in low-income (host) countries as opposed to their countries of origin [high-income nations] [66,67].

In the study by Ulvund and Mordal [66], students were reported to have experienced strong emotional reactions due to cultural differences. Students developed an openness to another culture, and a cultural knowledge base became a coping mechanism in a foreign culture. The international clinical placement experience broadened the perspectives of the students and caused them to embrace the diversities in abilities, personalities, and knowledge of inhabitants of the host country as well as those of their countries of origin. Evidence further indicated that the students developed interpersonal skills regarding working with people from diverse cultures [66].

Sundal and Ulvund [67] investigated nurses’ experiences after participating in an international clinical placement programme while remaining for one to three weeks in a paediatric ward. The students gained experiences and insight into further medical conditions unknown to them. They also assumed additional observer roles and hence performed minimal nursing procedures.

## Discussion

This scoping review reported on identified and analysed strategies to enhance clinical teaching and learning in undergraduate nursing education. Predominantly, studies from high-income countries featured, mainly from the United States, with few contributions from Asia and Africa. The review included a diverse population, primarily focusing on nursing students, with some studies incorporating nurse educators, teaching staff, preceptors, registered nurses, clinical associates, and nursing instructors. This range of participants provided a broad perspective on the various strategies employed in clinical nursing education. It is important to incorporate a range of individuals who may not necessarily be involved directly in clinical teaching and learning, such as administrators [68].

Most of the studies concentrated on exploring the experiences of nursing students, educators, and patients in terms of approaches and programmes intended to improve clinical education outcomes. Significant emphasis was placed on developing and implementing specific programmes and comparing various approaches within clinical education. Some studies proposed frameworks for clinical teaching and learning, highlighting the need for a structured approach in nursing education. Most research that evaluated the influence of their strategy to enhance clinical teaching and learning aligned with the lower levels of the Kirkpatrick evaluation model [69] indicated a shortage of outcome-based evaluations in this field. Longitudinal studies that emphasise measuring the impact or higher levels of the Kirkpatrick evaluation model should be conducted to provide plausible solutions to clinical teaching and learning in undergraduate nursing education. Seven strategies in terms of undergraduate nursing education were identified in the review of scholarly work.

First, scaffolding the nursing curriculum involves deliberate sequential revision of learning experiences from simple to complex and incorporates modern tools, such as smartphones and student dyads. The focus was on moving from simple to complex skills, ensuring that the curriculum responds effectively to the evolving demands of the nursing profession. Masava et al. [70], who recently reviewed approaches to scaffolding in higher education, discuss the need for an integrated multi-layered approach to emphasise the macro-, meso- and micro-elements of an educational programme. They highlight the need for the complexity of health sciences programmes when they argue for scaffolding to contribute to competence development among students when applied across all learning platforms, including the clinical learning environment.

In transformative teaching and learning approaches, experiential learning theories, peer-assisted learning, photovoice, clinical preceptor roles, and innovative approaches, such as the multiple intelligence teaching approach and iterative problem solving, were emphasised. These methods foster students’ competence, self-confidence, and adaptability in various clinical settings. Van Schalkwyk et al. [71] confirmed that health sciences students, including undergraduate nurses, are often exposed to unfamiliar learning environments. At the same time, they are expected to be active in-patient care. Fundamentally, educators must take advantage of these transformative learning environments through transformative teaching and learning to enhance clinical teaching and learning. For educators to apply transformative teaching approaches, the development of their skills related to the three pillars of academic medicine, i.e., skills related to the roles of teacher and educator, researcher and scholar, and administrator and leader, must be prioritised and enacted in transformative approaches, and they must be allowed to reflect on their current practice.

Simulation-based education was highlighted as a critical educational technique [72,73]. Various forms of simulation, including high-fidelity simulation, virtual simulation, and mass casualty incident simulation, were discussed [43,47,49]. Simulation addresses limitations in clinical settings and promotes teamwork, communication, critical thinking, and psychomotor skills among students. Literature reports on the utility of simulation-based education in undergraduate nursing compared to traditional clinical teaching and learning methods. However, the positive effect of simulation-based education is reported predominantly in high-income countries. Haerling et al. [74] concur by indicating that manikin-based simulation-based education is costly compared to traditional clinical education. This cost of establishing and integrating simulation-based education in undergraduate nursing education, especially in low-income settings, makes this type of education less attractive as a strategy to enhance clinical teaching and learning. Low-resource settings are in dire need of nurses. At the same time, they have limited economic and infrastructural resources to invest in nursing education, specifically for modern education strategies, such as simulation-based education. Simulation-based education needs to be contextualised based on available, inclusive, and sustainable resources.

Creating dedicated education units at clinical sites was proposed to facilitate authentic learning environments [50–52]. Such units could enhance students’ skills and knowledge, although resource constraints could pose challenges in some settings. Marcellus et al. [75] argue that dedicated education units are based on academic–practice partnerships, adaptability to diverse contexts, unit culture of educational excellence, responsive and supportive leadership, and clear roles and responsibilities. The literature is, however, inconclusive regarding the cost and benefits of establishing dedicated education units and their adoption in non-Western settings. Considerations on the structure and approach to integrating dedicated education units (DEUs) in undergraduate programmes must be prioritised, aligning with population-focused clinical teaching and learning.

The role of technology in clinical education was evident in the use of smartphones, instant messaging, mobile applications, virtual reality, augmented reality, virtual patient-based social learning, collaborative virtual teams, digital educational resources, and online problem-based learning. While these technological innovations offer vast benefits, they also present challenges in bridging theory–practice gaps and ensuring equitable access to technology.

Developing interprofessional teamwork and collaboration through approaches such as peer support, bearing witness, improving faculty–practice relationships, and interprofessional mentoring focuses on enhancing communication and building effective relationships among healthcare stakeholders. Interprofessional education has been heralded as a pivotal strategy to improve collaborative practice and health outcomes [76]. Khalili et al. [77], however, note that the adoption of interprofessional education (IPE) has been slow, especially in low-income countries, which are hampered by poor faculty development related to IPE, limited clinical examples of IPE, and the general lack of political will to invest in IPE. To contribute significantly to an increase in the training and number of nurses for the future, transformative approaches to education, such as interprofessional education, must be integrated into undergraduate curricula.

Lastly, integrative inter-country clinical experience was found to be beneficial in developing cultural awareness, competence, and sensitivity among nursing students [66,67]. International placements, particularly in low-resource settings, enriched students’ learning experiences by exposing them to diverse cultural and linguistic environments. Despite the insights provided by these findings, the limitations of the current review in terms of language and geographic scope suggest a need for more diverse and comprehensive research. This includes longitudinal studies evaluating clinical teaching and learning outcomes, and strategies that address the gap between theory and practice in nursing education globally. This review thus underscores the necessity of broadening the scope of research in clinical nursing education to prepare students better for the diverse challenges they will face in their professional careers.

## Conclusion

The current study employed a scoping review methodology guided by contemporary frameworks to describe what is known in the literature regarding clinical teaching and learning strategies to enhance undergraduate nursing education. The majority of these strategies were reported from the United States, with evidence of a paucity of research from low- and middle-income countries. In most of the articles reviewed, the longitudinal effect of the clinical teaching and learning strategies was not reported. The strategies identified could guide curriculum developers, programme organisers, nursing teachers, nursing students, and clinical instructors at clinical learning sites in streamlining the clinical teaching and learning of undergraduate nursing education. Implementing the key strategies identified in this review could improve clinical teaching and learning outcomes, which could culminate in the training of competent nurses. Implementation of some of the strategies may, however, be influenced by context-specific dynamics.

Based on the insight gained during this scoping review on clinical teaching and learning strategies, we recommend the following:

- the scaffolding of the nursing curriculum through the deliberate sequencing of clinical learning objectives and introducing the use of smartphones and student dyads in the foundational years of the nursing programme;
- employing transformative teaching and learning strategies using experiential learning theories and train teaching staff and clinical educators in andragogical approaches;
- creating well-resourced DEUs at clinical placement sites with the requisite expert personnel;
- integrating technological applications in clinical education as a supplementary learning resource for face-to-face teacher-learner interactions;
- intentional scheduling of joint clinical placement experiences among health professions students to lay the foundation of interprofessional collaboration; and
- planning inter-country clinical placement experiences for nursing trainees to develop their cultural competence for professional life.

Strategies to enhance clinical teaching and learning are useful in supporting reforms within undergraduate nursing education towards contributing to competent nurses for the future.

## Limitations

Notwithstanding the findings of this review, some limitations should be mentioned. Firstly, only studies with full-text articles were considered for inclusion, excluding studies with only abstracts. Secondly, the majority of the studies included used qualitative designs. This may limit the generalisability and validity of the themes, hence, the results of this review. This scoping review did not evaluate the quality and rigour of included studies. Another limitation is that only English search terms were used during the search. This resulted in the non-identification of studies published in other languages, which might have been relevant to this scoping review.

## Supporting information

S1 Table Articles included in the first-round screening. (PDF)

S2 Table Characteristics of the articles included in the scoping review. (PDF)
