# Supplementary material for: Strategies to enhance clinical teaching and learning in undergraduate nursing education: A scoping review
Source: PLoS One. 2025 Jun 10;20(6):e0305789. doi: 10.1371/journal.pone.0305789 (PMC12151355; doi:10.1371/journal.pone.0305789)
Supplement: S1 Table — (PDF) [file pone.0305789.s001.pdf]

S1 Table Article included in the first-round screening

NO	Title of Article	Included/Excluded	Reason for exclusion
1.	Education/clinical practice in primary health care, in Dakar (1998)	Excluded	Not within the time frame
2	A Canada-Bangladesh partnership for nurse education: case study (2010)	Excluded	Not within the time frame
3	A comparison of an international experience for nursing students in developed and developing countries (2000)	Excluded	Not within the time frame
4	A Nurse-Managed Advocacy Clinic in a Hispanic Senior Center (2014)	Excluded	Article not related to clinical teaching and learning
5	A reputational risk for the profession: Workplace violence toward nursing students (2020)	Excluded	Article not related to clinical teaching and learning
6	Advancing nursing scholarship: the Mozambique model (2017)	Excluded	Wrong population
7	Advancing nursing scholarship: the Mozambique model (2017)	Excluded	Duplicated
8	Advancing nursing scholarship: the Mozambique model. (2017)	Excluded	Duplicated
9	An approach to international education in primary health care (1988)	Excluded	Not within the time frame
10	An Australian mixed methods pilot study exploring students performing patient risk screening (2016)	Excluded	Wrong population
11	Attributional theory applied to a baccalaureate nursing community experience (1993)	Excluded	Not within the time frame
12	Bearing witness: to promote therapeutic effectiveness (2013)	Included	-
13	Cancer detection activities coordinated by nursing students in community health (1996)	Excluded	Not within the time frame
14	Clinical nurse specialist practicum in Nicaragua (2000)	Excluded	Not within the time frame
15	Clinical teaching of university-degree nursing students: are the nurses in practice in Uganda ready? (2021)	Excluded	No clear strategy indicated
16	Collaborating to optimize nursing students' agency information technology use. (2009)	Excluded	Not within the time frame
17	Collaborative Learning Using Nursing Student Dyads in the Clinical Setting (2013)	Included	-
18	Community health nursing in a nonclinical setting: service-learning outcomes of undergraduate students and clients (2004)	Excluded	Not within the time frame
19	Comparing Outcomes of Active Student and Observer Roles in Nursing Simulation (2019)	Included	-
20	COVID-19: Impact on undergraduate nursing education in Sri Lanka (2020)	Excluded	No clear strategy indicated
21	Developing Nursing Leadership Potential Through Education in a Low-Resource Setting (2021)	Excluded	Article not related to clinical teaching and learning

S1 Table Article included in the first-round screening

NO	Title of Article	Included/Excluded	Reason for exclusion
22	Development of a tool to assess students' perceptions of respectful maternity care (2022)	Excluded	Article not related to clinical teaching and learning
23	Emerging Role of Clinical Preceptors (CPs) at a Private University, Karachi, Pakistan (2017)	Included	-
24	Expanding urban learning experiences for non-traditional students (1999)	Excluded	Not within the time frame
25	Exploring factors affecting the facilitation of nursing students to learn paediatric pain management in Rwanda: A descriptive qualitative study (2022)	Excluded	Article not related to clinical teaching and learning
26	Exploring migration intention of nursing students in Nepal: A mixed-methods study (2018)	Excluded	Article not related to clinical teaching and learning
27	Exploring migration intention of nursing students in Nepal: A mixed-methods study (2018)	Excluded	Duplicated
28	Faculty Reflections on International Travel to Reach the Medically Underserved (2019)	Excluded	Article not related to clinical teaching and learning
29	HIV- and AIDS-related essential competencies for nurses in South Africa: Nurse educators and nurses in clinical practice identification (2018)	Excluded	Article not related to clinical teaching and learning
30	Instant messaging and nursing students' clinical learning experience (2018)	Included	-
31	Instant messaging and nursing students' clinical learning experience (2018)	Excluded	Duplicated
32	Interprofessional Mass Casualty Incident Simulation Design Protocol to Prepare Prelicensure Nursing Students to Respond to a Disaster (2017)	Included	-
33	Minorities in Nursing Education: Using Smartphones (2015)	Included	-
34	Nominal group process as an instructional method with novice community health nursing students (1988)	Excluded	Not within the time frame
35	Nursing students' experiences with simulation-based education as a pedagogic method in low-resource settings: A mixed-method study (2022)	Included	-
36	School: a clinical placement for community health nursing (1988)	Excluded	Not within the time frame
37	Self-assessed confidence of students on selected midwifery skills: Comparing diploma and bachelors programmes in one province of India (2018)	Excluded	Article not related to clinical teaching and learning
38	The impact of short term clinical placement in a developing country on nursing students: A qualitative descriptive study (2017)	Included	-
39	The impact of short term clinical placement in a developing country on nursing students: A qualitative descriptive study (2017)	Excluded	Duplicated

S1 Table Article included in the first-round screening

NO	Title of Article	Included/Excluded	Reason for exclusion
40	The influence of participating in an international clinical experience during baccalaureate nursing education on interprofessional collaboration and teamwork for new registered nurses (2015)	Included	-
41	Thinking in nursing education. Part I. A student's experience learning to think. (1999)	Excluded	Not within the time frame
42	Transforming experiences: nursing education and international immersion programs (2009)	Excluded	Not within the time frame
43	Using flash cards to engage Indonesian nursing students in reflection on their practice (2016)	Excluded	Wrong population
44	Using Polvika's model to create a service-learning partnership (2003)	Excluded	Not within the time frame
45	A collaborative approach to developing "learning synergy" in primary health care (2007)	Excluded	Not within the time frame
46	A partnership approach to the preparation of preceptors (2009)	Excluded	Not within the time frame
47	A program to provide resources and support for clinical associates (2013)	Included	-
48	A regional partnership to promote nursing instructor competence and confidence in simulation (2011)	Included	-
49	A strategy for maintaining student wellbeing (2015)	Excluded	Article not related to clinical education
50	A study of personal digital assistants to enhance Undergraduate Clinical Nursing Education (2005)	Excluded	Not within the time frame
51	A systematic review evaluating the impact of online or blended learning vs. face-to-face learning of clinical skills in undergraduate nurse education (2015)	Excluded	No clear strategy indicated
52	A Theoretical Framework to Underpin Clinical Learning for Undergraduate Nursing Students (2020)	Included	-
53	Academic nursing education guidelines: tool for bridging the gap between theory, research and practice (2003)	Excluded	Not within the time frame
54	Academic Outcome Measures of a Dedicated Education Unit Over Time: Help or Hinder? (2015)	Included	-
55	Adapting and Integrating Photovoice in a Baccalaureate Community Course to Enhance Clinical Experiential Learning (2015)	Included	-
56	Advancing nursing scholarship: the Mozambique model (2017)	Excluded	Duplicated
57	Advancing nursing scholarship: the Mozambique model (2017)	Excluded	Duplicated

S1 Table Article included in the first-round screening

NO	Title of Article	Included/Excluded	Reason for exclusion
58	Advancing nursing scholarship: the Mozambique model (2017)	Excluded	Duplicated
59	An exploration of peer-assisted learning in undergraduate nursing students in paediatric clinical settings: An ethnographic study (2018)	Included	-
60	An exploratory trial exploring the use of a multiple intelligences teaching approach (MITA) for teaching clinical skills to first year undergraduate nursing students (2015)	Included	-
61	An iterative approach to enhance the clinical learning experience in Macao nursing education (2019)	Included	-
62	Anxiety in student nurses in the clinical setting: a phenomenological study (2008)	Excluded	Not within the time frame
63	Are you man enough to be a nurse? The road less traveled (2011)	Excluded	Article not related to clinical teaching and learning
64	Arts-Based Learning Strategies in Clinical Postconference: A Qualitative Study (2018)	Excluded	No clear strategy indicated
65	Augmented Reality M-Learning to Enhance Nursing Skills Acquisition in the Clinical Skills Laboratory (2015)	Included	-
66	Bachelor of nursing students' experience of dialogue with nurse lecturers (2018)	Excluded	Article not related to clinical teaching and learning
67	Benefits and costs of integrating technology into undergraduate nursing programs (2005)	Excluded	Not within the time frame
68	Broken Communication in Nursing Can Kill: Teaching Communication Is Vital (2013)	Excluded	Article not related to clinical teaching and learning
69	Case-Based Web Learning Versus Face-to-Face Learning: A Mixed-Method Study on University Nursing Students (2016)	Excluded	Article not related to clinical teaching and learning
70	Case-Based Web Learning Versus Face-to-Face Learning: A Mixed-Method Study on University Nursing Students (2016)	Excluded	Duplicated
71	Clinical conferences--the key to successful experiential learning (1990)	Excluded	Not within the time frame
72	Clinical placement anxiety in undergraduate nursing students: A concept analysis (2020)	Excluded	No clear strategy indicated
73	Collaborative learning in the COVID-19 pandemic: A change to the delivery of undergraduate nursing education (2021)	Included	-
74	Defining Clinical Assessment Standards for Bachelor's-Prepared Nurses in Switzerland (2009)	Excluded	Not within the time frame

S1 Table Article included in the first-round screening

NO	Title of Article	Included/Excluded	Reason for exclusion
75	Developing an integrated approach to the assessment of student nurse competence using the Total Client Care (TCC) assessment tool (2020)	Excluded	No clear strategy indicated
76	Developing and integrating nursing competence through authentic technology-enhanced clinical simulation education: Pedagogies for reconceptualising the theory-practice gap (2019)	Excluded	No clear strategy indicated
77	Developing Leadership in Geriatric Education: An Annual Faculty Institute (1989)	Excluded	Not within the time frame
78	Development and psychometric testing of Holistic Clinical Assessment Tool (HCAT) for undergraduate nursing students (2016)	Excluded	No clear strategy indicated
79	Development and validation of a Clinical Assessment Tool for Nursing Education (CAT-NE) (2016)	Excluded	No clear strategy indicated
80	Educating for health service reform: clinical learning, governance and capability - a case study protocol (2016)	Excluded	Wrong population
81	Enablers of the interpersonal relationship between registered nurses and students on clinical placement: A phenomenological study (2021)	Excluded	No clear strategy indicated
82	Engaging Clinical Nurses in Quality Improvement Projects (2015)	Excluded	Article not related to clinical teaching and learning
83	Enhancing the Prelicensure Clinical Learning Experience: A Student-Centered Learning Approach (2022)	Excluded	No clear strategy indicated
84	ESP (enhancing strategic partnerships) (2004)	Excluded	Not within the time frame
85	Evaluation of Clinical Growth and Nursing Student Motivation in the Traditional Clinical Learning Environment (2018)	Excluded	No clear strategy indicated
86	Evaluation of strategies designed to enhance student engagement and success of indigenous midwifery students in an Away-From-Base Bachelor of Midwifery Program in Australia: A qualitative research study (2018)	Excluded	Wrong population
87	Evaluation of strategies designed to enhance student engagement and success of indigenous midwifery students in an Away-From-Base Bachelor of Midwifery Program in Australia: A qualitative research study (2018)	Excluded	Duplicated
88	Evaluation outcomes of a dedicated education unit in a baccalaureate nursing program (2012)	Included	-
89	Experiential learning in practice: An ethnographic study among nursing students and preceptors (2018)	Included	-

S1 Table Article included in the first-round screening

NO	Title of Article	Included/Excluded	Reason for exclusion
90	Exploring factors affecting the facilitation of nursing students to learn paediatric pain management in Rwanda: A descriptive qualitative study (2022)	Excluded	Duplicated
91	Exploring the challenges of clinical education in nursing and strategies to improve it: A qualitative study (2018)	Included	-
92	Exploring the environment of clinical baccalaureate nursing students' education in Iran; A qualitative descriptive study (2015)	Excluded	No clear strategy indicated
93	Factors in the clinical learning environment that influence caring behaviors of undergraduate nursing students: An integrative review (2022)	Excluded	Article not related to clinical teaching and learning
94	Faculty Perceptions of Simulation on Student Learning for Safe Clinical Nursing Practice (2013)	Excluded	No clear strategy indicated
95	Feature Article University and service sector collaboration for undergraduate psychiatric nursing education (2004)	Excluded	Not within the time frame
96	Final year undergraduate nursing students' experience of high-fidelity simulation: results of a survey (2022)	Included	-
97	From Student to Practicing Oncology Nurse: A Novel Collaboration to Create a Transition to Practice Program in Ambulatory Cancer Care (2016)	Included	-
98	Handheld computers in nursing education: PDA pilot project (2008)	Excluded	Not within the time frame
99	"I found it daunting": An exploration of educational needs and experiences of mental health student nurses working with children and adolescents with eating disorders (2020)	Excluded	Article not related to clinical teaching and learning
100	"I found it daunting": An exploration of educational needs and experiences of mental health student nurses working with children and adolescents with eating disorders (2020)	Excluded	Duplicated
101	Identification of a model masters degree in nursing for the community-based primary health care nurse practitioner (1994)	Excluded	Not within the time frame
102	Improving Active Collaborative Clinical Learning Through a Mobile Application for Undergraduate Nursing Students (2022)	Included	-
103	Improving clinical experiences for nursing students in nursing homes: An integrative literature review (2021)	Excluded	No clear strategy indicated
104	Innovative strategies: Increased engagement and synthesis in online advanced practice nursing education (2019)	Excluded	Wrong population

S1 Table Article included in the first-round screening

NO	Title of Article	Included/Excluded	Reason for exclusion
105	Interprofessional mentoring: enhancing students' clinical learning (2011)	Included	-
106	'It's complicated': Staff nurse perceptions of their influence on nursing students' learning. A qualitative descriptive study (2018)	Excluded	No clear strategy indicated
107	Learning clinical procedures through Internet visual resources: a qualitative study amongst undergraduate students (2015)	Excluded	Full-text not accessible
108	Learning experience of nursing students in a clinical partnership model: An exploratory qualitative analysis (2019)	Included	-
109	Learning With Laughter: Implementing Engaging Virtual Simulation During the COVID-19 Pandemic (2022)	Included	-
110	Lessons learned from developing, implementing, and evaluating a model of community-driven nursing (2001)	Excluded	Not within the time frame
111	Lessons Learned: Utilizing the NEWS Tool in High-Fidelity Simulation (2021)	Excluded	No clear strategy indicated
112	"My mentor didn't speak to me for the first four weeks": Perceived Unfairness experienced by nursing students in clinical practice settings (2018)	Excluded	No clear strategy indicated
113	Number of students in clinical placement and the quality of the clinical learning environment: A cross-sectional study of nursing and midwifery students (2022)	Excluded	No clear strategy indicated
114	Nursing interns' perception of clinical competence upon completion of preceptorship experience in Saudi Arabia (2018)	Excluded	Wrong population
115	Nursing student-patient relationship and related factors-A self-assessment by nursing students (2020)	Excluded	No clear strategy indicated
116	Nursing students' experience of clinical supervision and contributing factors in Jordan (2020)	Excluded	No clear strategy indicated
117	Nursing students' perceptions about clinical learning environment in Turkey (2016)	Excluded	No clear strategy indicated
118	Nursing students' perceptions of the qualities of a clinical facilitator that enhance learning (2017)	Excluded	No clear strategy indicated
119	Partnership working between a higher education institution and NHS TRUSTS: developing an acute and critical care module (2006)	Excluded	Not within the time frame
120	Peanuts, Popcorn, and Pediatrics: School-Based Carnival for Health Promotion (2021)	Excluded	Article not related to clinical teaching and learning
121	Perceptions and evaluation regarding clinical teaching among nursing students in a selected nursing college in Selangor (2016)	Excluded	No clear strategy indicated

S1 Table Article included in the first-round screening

NO	Title of Article	Included/Excluded	Reason for exclusion
122	Power dynamics in the student-teacher relationship in clinical settings (2017)	Excluded	No clear strategy indicated
123	Precepted and non-precepted senior baccalaureate nursing students' understanding of basic leadership principles (1996)	Excluded	Not within the time frame
124	Preceptorship: a viable alternative clinical teaching strategy? (1988)	Excluded	Not within the time frame
125	Preparing advanced practice nurses for clinical decision making in specialty practice (1997)	Excluded	Not within the time frame
126	Preparing nursing students for the future: an innovative approach to clinical education (2013)	Included	-
127	Psychometric properties of the clinical learning environment, Supervision and Nurse Teacher scale (CLES+T) for undergraduate nursing students in Hong Kong (2021)	Excluded	No clear strategy indicated
128	Putting the 'patient' back into patient-centred care: An education perspective (2013)	Excluded	Article not related to clinical teaching and learning
129	Readiness for practice in undergraduate nursing students during the COVID-19 pandemic: a cross-sectional study (2022)	Excluded	No clear strategy indicated
130	Recognizing, responding to and reporting patient deterioration: transferring simulation learning to patient care settings (2012)	Included	-
131	Reflective debriefing to promote novice nurses' clinical judgment after high-fidelity clinical simulation: a pilot test (2013)	Excluded	Wrong population
132	Sensitive Situations in a Nurse Residency Program: Balancing Confidentiality With Meaningful Solutions (2021)	Excluded	Article not related to clinical teaching and learning
133	Student nurses' learning processes in interaction with psychiatric patients: A qualitative investigation (2011)	Excluded	Article not related to clinical teaching and learning
134	Student-Guided, Theme-Based Postclinical Conference to Enhance Student Involvement in Clinical Learning (2018)	Included	-
135	Teaching population health and community-based care across diverse clinical experiences: integration of conceptual pillars and constructivist learning (2014)	Excluded	Wrong population
136	The Bucket List: a service-learning approach to community engagement to enhance community health nursing clinical learning (2011)	Excluded	Article not related to clinical teaching and learning
137	The Clinical Pause: An Augmented Approach to Simulation Debriefing in Nursing Education (2022)	Included	-

S1 Table Article included in the first-round screening

NO	Title of Article	Included/Excluded	Reason for exclusion
138	The effect of peer support approach on communication skills of nursing students in pediatric clinical setting (2021)	Included	-
139	The effectiveness of the virtual patient-based social learning approach in undergraduate nursing education: A quasi-experimental study (2022)	Included	-
140	The effects of simulation on nursing students' critical thinking scores: a quantitative study (2009)	Excluded	Not within the time frame
141	The experiences of undergraduate Assistants in Nursing (AIN) (2012)	Excluded	Wrong population
142	The 'five rights' of clinical reasoning: an educational model to enhance nursing students' ability to identify and manage clinically 'at risk' patients (2010)	Excluded	Not within the time frame
143	The good clinical nursing educator and the baccalaureate nursing clinical experience: attributes and praxis (2008)	Excluded	Not within the time frame
144	The impact of clinical simulation on learner self-efficacy in pre-registration nursing education (2010)	Excluded	Not within the time frame
145	The impact of utilizing high-fidelity computer simulation on critical thinking abilities and learning outcomes in undergraduate nursing students (2004)	Excluded	Not within the time frame
146	The Leader-Clinician Advocate in Advancing Student Clinical Learning. (English) By: Bleich MR, Journal of continuing education in nursing (2017)	Excluded	No clear strategy indicated
147	The nurse-patient communication: voices from nursing students (2016)	Excluded	Article not related to clinical teaching and learning
148	The nurse-patient communication: voices from nursing students (2016)	Excluded	Duplicated
149	The reality of virtual reality at a South African university during the COVID-19 pandemic (2021)	Included	-
150	Undergraduate nursing students' experience of clinical supervision (2018)	Excluded	No clear strategy indicated
151	Undergraduate nursing students' experience of clinical supervision (2018)	Excluded	Duplicated
152	Underrepresented Ethnic Minority Nursing Students' Perceptions of Factors That Enhanced or Hindered Their Self-Efficacy (2022)	Excluded	Article not related to clinical teaching and learning
153	University and service sector collaboration for undergraduate psychiatric nursing education (2004)	Excluded	Not within the time frame
154	University and service sector collaboration for undergraduate psychiatric nursing education (2004)	Excluded	Duplicated

S1 Table Article included in the first-round screening

NO	Title of Article	Included/Excluded	Reason for exclusion
155	Work-engaged nurses for a better clinical learning environment: a ward-level analysis (2016)	Excluded	Article not related to clinical teaching and learning
156	Development of a tool to assess students' perceptions of respectful maternity care (2022)	Excluded	Duplicated
157	Experiences after international clinical placement as nursing students in a paediatric ward (2023)	Included	-
158	Exploring factors affecting the facilitation of nursing students to learn paediatric pain management in Rwanda: A descriptive qualitative study (2022)	Excluded	Duplicated
159	"No PBL is better than online PBL": Qualitative exploration regarding the perceived impact of online problem-based learning on nursing and medical students' learning during COVID-I9 lockdown (2023)	Included	-
160	Nursing students' experiences with simulation-based education as a pedagogic method in low-resource settings: A mixed-method study (2022)	Excluded	Duplicated
161	Retention in nursing education and scholarship programs: Survival analysis of the Veterans Health Administration National Nursing Education Initiative Data (2023)	Excluded	Article not related to clinical teaching and learning
162	Student nurse education and preparation for palliative care: A scoping review (2023)	Excluded	Article not related to clinical teaching and learning
163	Training Student Volunteers as "Community Resource Navigators" to Integrate Health and Social Care in Primary Care (2022)	Excluded	Article not related to clinical teaching and learning
164	Co-creating digital educational resources to enhance quality in student nurses' clinical education in nursing homes: Report of a co-creative process (2023)	Included	-
165	Enhancing the Prelicensure Clinical Learning Experience: A Student-Centered Learning Approach (2022)	Excluded	Duplicated
166	Exploring factors affecting the facilitation of nursing students to learn paediatric pain management in Rwanda: A descriptive qualitative study (2022)	Excluded	Duplicated
167	Factors in the clinical learning environment that influence caring behaviors of undergraduate nursing students: An integrative review (2022)	Excluded	Duplicated
168	Final year undergraduate nursing students' experience of high-fidelity simulation: results of a survey (2022)	Excluded	Duplicated
169	Improving Active Collaborative Clinical Learning Through a Mobile Application for Undergraduate Nursing Students (2023)	Excluded	Duplicated

S1 Table Article included in the first-round screening

NO	Title of Article	Included/Excluded	Reason for exclusion
170	Improving Active Collaborative Clinical Learning Through a Mobile Application for Undergraduate Nursing Students (2023)	Excluded	Duplicated
171	Learning With Laughter: Implementing Engaging Virtual Simulation During the COVID-19 Pandemic (2022)	Excluded	Duplicated
172	Number of students in clinical placement and the quality of the clinical learning environment: A cross-sectional study of nursing and midwifery students (2022)	Excluded	Duplicated
173	Readiness for practice in undergraduate nursing students during the COVID-19 pandemic: a cross-sectional study (2022)	Excluded	Duplicated
174	Replacing the Pediatric Traditional Clinical Experience with High-Fidelity Simulation in an Associate Degree Nursing Program (2022)	Included	-
175	Teaching Strategies for Health Advocacy for Undergraduate Nursing Students: A Scoping Review	Excluded	Article not related to clinical teaching and learning
176	The Clinical Pause: An Augmented Approach to Simulation Debriefing in Nursing Education (2022)	Excluded	Duplicated
177	The effectiveness of the virtual patient-based social learning approach in undergraduate nursing education: A quasi-experimental study (2022	Excluded	Duplicated
178	Underrepresented Ethnic Minority Nursing Students' Perceptions of Factors That Enhanced or Hindered Their Self-Efficacy	Excluded	Duplicated
