# Supplementary material for: Strategies to enhance clinical teaching and learning in undergraduate nursing education: A scoping review
Source: PLoS One. 2025 Jun 10;20(6):e0305789. doi: 10.1371/journal.pone.0305789 (PMC12151355; doi:10.1371/journal.pone.0305789)
Supplement: S2 Table — (PDF) [file pone.0305789.s002.pdf]

S2 Table Characteristics of articles included in the scoping review

No	Author/ Year	Country	Title of the study	Design	Purpose of the study	Sample Size	Population	Name of strategy	Main Findings	Recommendations
1	Hunt et al. (2013) (37)	USA	A program to provide resources and support for clinical associates	Not reported	To describe the development of an orientation course and ongoing resources and support designed to facilitate the transition into a clinical instructor role for registered nurses who teach in a baccalaureate nursing program.	Not reported	Clinical Associates	The Clinical Associate Resources and Support (CARS) orientation program	**Orientation of Clinical Associates on:**<br>-Overview of the curriculum, including mission, philosophy, and outcome objectives of the school of nursing.<br>-Effective clinical teaching strategies, including pre and post-clinical conferences, critical thinking activities, time management, and cluster teaching<br>-Faculty-clinical associate mentoring relationship<br>-Faculty-clinical associate communication (channels, benefits, frequency)<br>-How to use student evaluation, recommendation process for referral, and remediation processes for students whose performance is unsatisfactory and unsafe in the clinical setting.<br>-Faculty expectations for care planning<br>-General student policies and procedures regarding absences,	Not reported

S2 Table Characteristics of articles included in the scoping review

No	Author/ Year	Country	Title of the study	Design	Purpose of the study	Sample Size	Population	Name of strategy	Main Findings	Recommendations
									lateness, professional appearance, student engagement, and confidentiality.<br><br>-Guidance on difficult student behaviour or uncooperative staff and suggestions for handling these situations.<br><br>-Discussions on clinical sites and requirements specific to the roles of educator and student.<br><br>-General employment information such as pay scale and frequency of payment.<br><br>-Networking session to share experiences in difficulties in the clinical setting and how they were handled, methods of linking classroom content to individual patient care, and innovative clinical teaching strategies.<br><br>-Course syllabus with weekly classroom and clinical objectives were given to each clinical associate.<br><br>-Clinical associates were granted access to a web-based portal as a	

S2 Table Characteristics of articles included in the scoping review

No	Author/ Year	Country	Title of the study	Design	Purpose of the study	Sample Size	Population	Name of strategy	Main Findings	Recommendations
									source of ongoing resources and support.	
2	Coleman et al. (2011) (48)	USA	A regional partnership to promote nursing instructor competence and confidence in simulation	Not reported	To describe a regional academic partnership between nursing programs and service partners in a 13-county region in the Texas Gulf Coast that used a train-the-trainer model and other activities to promote simulation.	Nursing instructors (n=263)	Nursing instructors from associate degree in nursing/ bachelor of science in nursing, and hospital staff development programs	Clinical simulation	-High cost of manikins/equipment<br>-Increased development of simulation laboratories<br>-Lack of adequately educated and trained laboratory administrators, technical support personnel<br>-Increased use of simulation as a pedagogy<br>-Increased purchase of manikins	Not reported
3	Landers et al. (2020) (31)	Ireland	A theoretical framework to underpin clinical learning for undergradu	Not reported	To propose a theoretical framework to underpin clinical learning for students as	Not reported	Not reported	Steinaker and Bell (1979)	Steinaker and Bell's Taxonomy<br>-This model is grounded in experiential learning theory and underpinned by constructivist conceptions of teaching and learning.	The authors suggest that educationalists draw on

S2 Table Characteristics of articles included in the scoping review

No	Author/ Year	Country	Title of the study	Design	Purpose of the study	Sample Size	Population	Name of strategy	Main Findings	Recommendations
			ate nursing students		they progress through the clinical components of an undergraduate nursing educational program.			taxonomy	-Constructivist learning is recognized as an approach to teaching that focuses more on deep than surface learning and equates more with professional competence.<br>-This taxonomy is an example of a tool comprising teaching strategies for planning, sequencing, implementing, and particularly evaluating the experience of teaching and learning.<br>-Steinaker and Bell (1979) considered the learning process as passing through five sequential levels: (a) exposure, (b) participation, (c) identification, (d) internalization, and (e) dissemination.<br>-The experiential taxonomy proposes a sequence to the learning and teaching activities, which begins when a learner is first exposed to a learning experience and continues to the point where the learner has internalized the experience and is able to disseminate it to others.<br>-For the undergraduate student, the first four steps of the taxonomy are considered appropriate, with the	other learning theories to guide the educational process depending on the needs of the leaner and the clinical learning situation.

S2 Table Characteristics of articles included in the scoping review

No	Author/ Year	Country	Title of the study	Design	Purpose of the study	Sample Size	Population	Name of strategy	Main Findings	Recommendations
									fifth step considered more suitable for the graduate nurse. The steps are intrinsically linked but rely greatly on the preceptor/mentor to encourage the learner to participate in an experience.<br><br>-During the exposure stage, the preceptor/mentor leads the activity using audio or visual materials, engages in questioning, and/or performs activities to stimulate interest.<br><br>-The second stage, participation, is dominated by the learner's physical and mental activity with the preceptor/mentor providing supportive feedback during the learning activity, to intervene when obstacles are encountered and to reveal participant learning that should result from successful completion of the exercise.<br><br>-The next stage, identification, emanates from the learner's emotional attachment and intellectual absorption during which they become cognizant of new	

S2 Table Characteristics of articles included in the scoping review

No	Author/ Year	Country	Title of the study	Design	Purpose of the study	Sample Size	Population	Name of strategy	Main Findings	Recommendations
									knowledge and acquired skills. The student then seeks more learning resources and learning opportunities and becomes more self-directed.<br>-As progression from identification to internalization develops, the preceptor/ mentor moderates but sustains learner engagement, identifies assignments that are progressively more difficult or have less obvious solutions, and engages in more advanced questioning about new learning and generalizations being formed by the experience.<br>-The taxonomy is presented as a practical medium for itemizing the nursing activities novices are exposed at the initiation of clinical experience to the clinical competencies expected of student nurses on completion of their undergraduate educational programs. The levels within the taxonomy also provide guidance to the preceptor/mentor when assessing the progression of the student through each clinical placement and at the end of each year of the program.	

S2 Table Characteristics of articles included in the scoping review

No	Author/ Year	Country	Title of the study	Design	Purpose of the study	Sample Size	Population	Name of strategy	Main Findings	Recommendations
									-To complement Benner's framework, the authors also propose the addition of Steinaker and Bell's (1979) experiential as a practical tool to enable preceptors/mentors to determine students' achievements/levels of learning at certain junctures during their 4-year undergraduate education program.	
4	Smyer et al. (2015) (52)	USA	Academic outcome measures of a dedicated education unit over time: help or hinder?	Longitudinal quasi-experimental repeated measures design to compare students in the DEU versus those in a traditional clinical setting.	To examine outcomes over time and compare students placed on a DEU during semester two with students placed on a traditional clinical unit to determine if the DEU helped or hindered students achieving	(n=144)	Students enrolled in an undergraduate program	Dedicated Education Unit	No significant differences were found among these 4 cohorts of participants of DEU and traditional clinical setting	More longitudinal studies are needed to compare student academic outcomes in DEUs versus a traditional clinical model. Future studies

S2 Table Characteristics of articles included in the scoping review

No	Author/ Year	Country	Title of the study	Design	Purpose of the study	Sample Size	Population	Name of strategy	Main Findings	Recommendations
					academic outcomes.					should focus on the role of the clinical instructor and academic faculty member in the DEU model.
5	Gallagher et al. (2015) (35)	USA	Adapting and integrating photovoice in a baccalaureate community course to enhance clinical experiential learning	Qualitative research method	To describe the adaptation and incorporation of a qualitative research method photovoice as a strategy to enhance the traditional windshield survey and key informant interviews in students'	(n=10)	A community clinical group of nursing students in the Bachelor of Science in Nursing program	Photovoice	-the students liked that they could use the photographs as a reference point during their critical dialogue and when developing their community nursing intervention.<br>- We were also able to refer to them later when writing the community paper.<br>- the photographs allowed them to have a holistic view of the community.<br>-Student felt it was time-consuming, indicating a lack of understanding of the purpose of the activity.	Not reported

S2 Table Characteristics of articles included in the scoping review

No	Author/ Year	Country	Title of the study	Design	Purpose of the study	Sample Size	Population	Name of strategy	Main Findings	Recommendations
					community health assessment project.				Perhaps using	
6	Carey et al. (2018) (33)	United Kingdom	An exploration of peer-assisted learning in undergraduate nursing students in paediatric clinical settings: An ethnographic study	Qualitative ethnographic approach	To explore peer-assisted learning (PAL) in undergraduate nursing students, studying children's health, in paediatric clinical settings.	(n=17)	The students were purposefully selected from a range of first, second and third-year paediatric nursing students enrolled on a three-year undergraduate nursing degree	Peer-assisted learning (PAL)	-Peers as facilitators to develop learning when engaging in PAL with peers acting as informal facilitators of learning towards their peer colleagues.<br><br>Peers engage in informal teaching support to offer advice and guidance to other peer colleagues, which are easier to remember.<br>- Questioning enabled peer pairs to openly engage and confirm their understanding of various clinical tasks with their peer colleagues.<br><br>- peer pairs plan and relate clinical and academic assessments assigned by the university<br><br>-They discuss what competencies they believe relates to practice and how they can relate this to their	To explore other fields of nursing to determine similarities and further influences of PAL amongst nursing students.

S2 Table Characteristics of articles included in the scoping review

No	Author/ Year	Country	Title of the study	Design	Purpose of the study	Sample Size	Population	Name of strategy	Main Findings	Recommendations
							programme		current placement<br><br>-Working Together to Develop Clinical Practice and Deliver Care<br>- peer interactions shape the development of the students' own clinical practice. They discuss the role of the student nurse in providing the parent with advice and guidance to ensure effective health promotion.<br><br>-Students demonstrated learning and development, through positive PAL interaction, within clinical practice and planning of patient care.<br><br>Within their pairings, students would often share in the learning experience of implementing and providing care for patients when engaging in PAL.	

S2 Table Characteristics of articles included in the scoping review

No	Author/ Year	Country	Title of the study	Design	Purpose of the study	Sample Size	Population	Name of strategy	Main Findings	Recommendations
									-Prior to undertaking these tasks students would establish the role they would like to perform.<br>-This approach was widely accepted by multiple pairs of students.<br>nurse.<br>- Positive Support and interaction from peers to enhance networking and develop a working structure.<br>- Students, when engaging in PAL, often used each other to navigate the clinical environment, especially in the early stages of a new placement.<br>-The engagement of PAL by both junior and senior peers highlighted a culture whereby they would feel confident to discuss openly structure of the clinical area and consider where they fit into this environment as student nurses<br>-Peers seemed to work well together in their pairings when engaging in PAL.	

S2 Table Characteristics of articles included in the scoping review

No	Author/ Year	Country	Title of the study	Design	Purpose of the study	Sample Size	Population	Name of strategy	Main Findings	Recommendations
									-Students were open to the opinions of their peer colleagues when engaging in shared learning and networking.<br><br>Networking also extended to opportunities for social interactions in<br>which students could offload stress and seek emotional support from their peer colleagues.	
7	Sheahan et al.<br>(2015) (38)	Ireland	An exploratory trial exploring the use of a multiple intelligences teaching approach (MITA) for teaching clinical skills to first year undergraduate	A Randomised Control Trial	To assess whether MITA is an effective method of teaching clinical skills to first year undergraduate nursing students.	(n=90)	All students enrolled on a first year BSc nursing degree were eligible for inclusion.	Multiple intelligences teaching approach (MITA)	Index of learning:<br>The majority of the participants preferred sensing as a style of learning.<br>Least learning style was intuition.<br>The highest multiple intelligence is interpersonal intelligence.<br>Multiple Intelligence Development Assessment Scale:<br>The highest MI assessment preference was interpersonal intelligence, and the weakest was naturalistic intelligence	The cost-effectiveness of MITA should be addressed in future studies.

S2 Table Characteristics of articles included in the scoping review

No	Author/ Year	Country	Title of the study	Design	Purpose of the study	Sample Size	Population	Name of strategy	Main Findings	Recommendations
			nursing students						Multiple Intelligence (MI) Assessment Preferences:<br>Most students preferred practical examinations and multiple-choice questions as assessment methods.<br>Positive features of the Multiple Intelligence Teaching Approach (MITA) Enablement of clinical skills learning.<br>MITA is a diverse learning method.<br>Approachable instructors and the use of music helped to reduce stress (environmental factors).<br>Negative features<br>Lack of practice time	
8	Liu et al.<br>(2019) (39)	Macau China	An iterative approach to enhance the clinical learning experience	A prospective cohort research design.	To reduce or eliminate the identified stressors and to enhance nursing	First year (n= 62); second year (n =54);	The participants included all full-time	Iterative problem -solving approach.	**Problem 1:** Students are stressed because of lacking confidence in the clinic	Not reported

S2 Table Characteristics of articles included in the scoping review

No	Author/ Year	Country	Title of the study	Design	Purpose of the study	Sample Size	Population	Name of strategy	Main Findings	Recommendations
			in Macao nursing education		students' clinical experience through the implementation of iterative problem-solving approach.	third year (n=50); fourth-year (n=48).	teaching staff in a nursing program and one cohort of nursing students who enrolled in the nursing program in 2014.		**Initial solution(s)**: Increasing compulsory practice hours in the school laboratory<br>**2nd Refine solution(s)**<br>Inviting teaching staff or senior student helpers to supervise students' practice in the laboratory<br>**3rd Refine solution(s)**<br>Setting up a "Rainbow family" in which each freshman is paired with one or two senior nursing students as his/her mentor(s)<br>**Problem 2:** Students are stressed because of fearing mistakes in clinical placement<br>**Initial solution(s):** Increasing compulsory practice hours in the school laboratory<br>**2nd Refine solution(s)**<br>Limiting the number of students under each preceptor.	

S2 Table Characteristics of articles included in the scoping review

No	Author/ Year	Country	Title of the study	Design	Purpose of the study	Sample Size	Population	Name of strategy	Main Findings	Recommendations
									Organising sharing meetings with outstanding senior students<br>**3rd Refine solution(s)**<br>Conducting special workshops for students at the beginning of each academic year. The topics include stress management, patient safety, infectious disease protection and prevention, etc.<br>**Problem 3:** Students are stressed because of workload and overwhelmed by responsibility<br>**Initial solution(s):** Simply reducing students' assignments<br>**2nd Refine solution(s)**<br>Integrating the assignments from both clinic preceptors and school faculty<br>**3rd Refine solution(s)**<br>-Employing a team-based learning (TBL) strategy for students to identify caring issues or cases in the	

S2 Table Characteristics of articles included in the scoping review

No	Author/ Year	Country	Title of the study	Design	Purpose of the study	Sample Size	Population	Name of strategy	Main Findings	Recommendations
									ward and to finish reports and evaluations as a team<br><br>-Briefing and negotiating with clinical preceptors or head nurses that students<br><br>cannot be used as 'extra staff' and cannot be given a level of responsibility beyond their experience level<br><br>**Problem 4:** Students are stressed because of the differences between what learned and real clinical practice<br><br>**Initial solution(s):** Briefing all preceptors before and after students' clinical placement in each semester<br><br>**2nd Refine solution(s)**<br><br>-Briefing and debriefing all preceptors before and after students' clinical placement in each semester.<br><br>-Updating the clinical placement handbook in each academic year<br><br>**3rd Refine solution(s)**	

S2 Table Characteristics of articles included in the scoping review

No	Author/ Year	Country	Title of the study	Design	Purpose of the study	Sample Size	Population	Name of strategy	Main Findings	Recommendations
									Conducting a clinical preceptor training program, which lets the preceptors know exactly what students learned in school and improves their precepting skills	
9	Garrett and Jackson (2015) (56)	Canada	Augmented reality m-learning to enhance nursing skills acquisition in clinical skills laboratory	Mixed method	To report on a pilot research project designed to explore if new mobile augmented reality (AR) technologies have the potential to enhance the learning of clinical skills in the lab.	Nursing students (n=160) and their instructors	Undergraduate Nursing students and instructors	Mobile augmented reality (AR) technologies	**Student perspectives**<br>-Most students indicated learning resources reflecting high technical quality<br>The majority agreed that AR resources were focused on specific skills<br>-Difficulty level of resources as appropriate<br>-Resources reflected contemporary knowledge and practices<br>-Resources easy to use.<br>-AR items were well organised in a consistent and logical fashion that was easy to use and follow.<br>-Access to learning material anywhere	Further integration and evaluative work with AR technologies are necessary to establish the practical value of AR in the clinical skills lab and beyond.

S2 Table Characteristics of articles included in the scoping review

No	Author/ Year	Country	Title of the study	Design	Purpose of the study	Sample Size	Population	Name of strategy	Main Findings	Recommendations
									-Resources helped in learning the required skills and knowledge<br>-Resources provided additional information that will help students in practice.<br>-promote reflexive review<br>-Some students felt the AR was a gimmick and did not support learning.<br>Student preferred media:<br>The majority of the students preferred video (81%) resources.<br>Text and PDF resources were less preferred<br>Internally created videos because the external videos were confusing<br>**-Instructor perspectives on AR implementation and value:**<br>-Most instructors agreed that AR resources would help students learn skills, provided useful additional information and preferred video-based AR resources.	

S2 Table Characteristics of articles included in the scoping review

No	Author/ Year	Country	Title of the study	Design	Purpose of the study	Sample Size	Population	Name of strategy	Main Findings	Recommendations
									-Instructors were divided on the ease of use<br>**Technical issues, training and replacement of demonstrations**<br>-difficulty scanning and downloading materials, internet connection issues, slow internet speed, small phone screens, lack of access to a smartphone, instability of application<br>**Suggestions from students to improve the AR in the lab**<br>-More training of students and instructors<br>-provide more AR resources/videos<br>-Broader exposure of the university community to the AR resource.<br>-Students do not want videos to replace demonstrations in the lab<br>**Future consideration**<br>Geospatial metadata (geotagging) for practice site information	
10	Minden (2013) (62)	USA	Bearing witness to promote	Not reported	To describe an educational approach used	(n=300)	Nursing students	Bearing Witness	This approach helped students to:	Not reported

S2 Table Characteristics of articles included in the scoping review

No	Author/ Year	Country	Title of the study	Design	Purpose of the study	Sample Size	Population	Name of strategy	Main Findings	Recommendations
			therapeutic effectiveness		in an undergraduate nursing clinical course to teach multiple intersecting, subtle, and nuanced intra- and interpersonal microabilities that underpin therapeutic effectiveness.				-Understand the conditions of their patients.<br>-Learn the components of skillful interviewing through observation.<br>-Appreciate teamwork/collaboration, support for one another and group cohesion. Not reported<br>-Develop their competence.<br>-Learn to co-lead, delegate, and direct.	
11	Thomson et al. (2021) (58)	New Zealand	Collaborative learning in the COVID-19 pandemic: a change to the delivery of undergraduate nursing education	Not reported	To explore an innovative solution to create a change in the delivery of undergraduate nursing education	Not reported	Not provided	Collaborative learning in a virtual team	-the academic team's motivation, deep understanding of nursing pedagogy, and experience as registered nurses, along with positivity and teamwork, were key drivers to enable the timely continuation of nursing students' learning within this course.<br>-it enhanced teamwork, demonstration of leadership, and relevant and applicable knowledge,	Not provided

S2 Table Characteristics of articles included in the scoping review

No	Author/ Year	Country	Title of the study	Design	Purpose of the study	Sample Size	Population	Name of strategy	Main Findings	Recommendations
									emphasising work readiness as future registered nurses.<br><br>-The implication for nursing education is that an appropriate learning experience can be substituted for a clinical learning experience in exceptional times and circumstances.<br><br>- Enhanced clinical skills, namely venepuncture, vaccination, and the unique opportunity for nursing students to gain clinical experiences at Managed Isolation Quarantine Facilities (MIQF).<br><br>-The disciplinary team established a safe and supportive learning environment for nursing students through procedures and protocols.<br><br>- Experience has led to employment for current graduates of the BN programme.	
12	Austria et al.<br><br>(2013) (30)	USA	Collaborative learning using nursing student dyads in the	Qualitative	To explore student and patient experiences and perspectives	(n=11)	Baccalaureate nursing students	Collaborative learning.	**Positive outcomes of the strategy**<br><br>-Cognitive processing and decision-making become a shared responsibility.	Not reported

S2 Table Characteristics of articles included in the scoping review

No	Author/ Year	Country	Title of the study	Design	Purpose of the study	Sample Size	Population	Name of strategy	Main Findings	Recommendations
			clinical Setting		of collaborative learning when student peer dyads are used for clinical instruction in the hospital setting.				-Working together served as a support base for students.<br>-This learning approach decreased anxiety among students.<br>-The confidence of students increased.<br>-Increase in the confidence nursing staff have in the students as they work in pairs.<br>-Completion of clinical tasks is done more efficiently.<br>-Increased patient satisfaction.<br>-Patients remarked that they received quality nursing care.<br>-Patients (learning apparatus) and clinical instructors (provide direction) supported students' learning.<br>**Negative outcomes of the strategy**<br>-Patients are overwhelmed, which leads to compromised health.<br>-Longer time for task completion.	

S2 Table Characteristics of articles included in the scoping review

No	Author/ Year	Country	Title of the study	Design	Purpose of the study	Sample Size	Population	Name of strategy	Main Findings	Recommendations
									-Task sharing led to missed earning opportunities due to the negotiation of the tasks.<br>-Conflict among student dyad due to dominance of one student performing most tasks.<br>**Consideration for curriculum**<br>Students suggested that peer dyads are most appropriate in the early semesters of the nursing programme.	
13	Bates et al. (2019) (43)	USA	Comparing outcomes of active student and observer roles in nursing simulation	A quasi-experimental study was conducted	To compare any differences between an observer role and active nursing role on student anxiety and learning outcomes in HFS.	(n=132)	Undergraduate nursing students.	High-fidelity simulation	-Increased pre-simulation anxiety and decreased post-simulation anxiety<br>-Improved students' ability to provide patient care<br>-Improve student problem-solving abilities<br>-Improve students' self-confidence in clinical practice,<br>-Ability to collaborate with members of the healthcare team and peers	Not reported

S2 Table Characteristics of articles included in the scoping review

No	Author/ Year	Country	Title of the study	Design	Purpose of the study	Sample Size	Population	Name of strategy	Main Findings	Recommendations
14	Dias et al. (2017)<br>(36)	Pakistan	Emerging role of clinical preceptors (CPs) at a private University, Karachi, Pakistan	Qualitative design	To understand the emerging role of the CPs in the undergraduate nursing program at a private university.	Not reported	Nursing administration both at School of nursing and Nursing Services, students, co-faculty.	Improving clinical preceptor roles	Students reported that:<br>-Clinical preceptors (CPs) knowledge and experience helped discharge their roles.<br>-Non-threatening attitudes of CPs reduced anxiety in students.<br>-Involvement of CPs in the clinical education of students for the period of study (4 years) enhanced students' self-confidence to do nursing procedures.<br>-CPs use an adult approach when handling clinical scenarios.<br>-CPs employed various clinical teaching methods (supervised practice session demonstrations and return demonstrations).<br>-CPs call students to discuss new learning opportunities or unique cases that may surface.<br>-CPs led discussions on cases at post-conferences.<br>-CPs long-standing good working relationship with staff made it easier for students to collaborate and	-Formal training in clinical teaching and feedback should be provided to CPs.<br>-CPs should be given course modules ahead of the clinical,<br>- Shadowing of preceptors on clinical by senior teaching staff for at

S2 Table Characteristics of articles included in the scoping review

No	Author/ Year	Country	Title of the study	Design	Purpose of the study	Sample Size	Population	Name of strategy	Main Findings	Recommendations
									communicate effectively with the healthcare team in the unit.<br>**Report from Faculty**<br>CPs were receptive to taking assignments and initiatives.<br>CPs sought guidance whenever necessary.<br>CPs accepted delegated duties.<br>CPs were seen by faculty to be competent.<br>**Report from Administration**<br>Nursing administration thinks hiring CPs is a good initiative.<br>Hiring of CPs meets the requirements of regulatory bodies and is cost-effective.<br>**Challenges the clinical preceptorship strategy reported by students, co-faculty and administration**<br>-Students and co-faculty reported that:	least one semester before they independently supervise students.<br>-Mentorship should be provided to the CPs on an ongoing basis.<br>-CPs should also be evaluated and given feedback on their

S2 Table Characteristics of articles included in the scoping review

No	Author/ Year	Country	Title of the study	Design	Purpose of the study	Sample Size	Population	Name of strategy	Main Findings	Recommendations
									There were hitches in integrating theory into practice in the clinical setting.<br>-Provision of timely and effective feedback.<br>Co-faculty reported that:<br>CPs had difficulty in keeping anecdotal records of the students and dealing with difficult students<br>CPs work output revealed their lack of experience and planned mentorship.	performance.
15	Rhodes et al.<br>(2012) (50)	USA	Evaluation outcomes of a dedicated education unit in a baccalaureate nursing program	Mixed-method	To evaluate the implementation of a DEU and to better understand the benefits and degree of satisfaction that educating student nurses on a DEU provides.	Students (n=85), faculty (n=4), and DEU nurses (n=45)	Students, faculty, and DEU nurses	Dedicated education unit (DEU)	-All students (100%) were happy with the DEU<br>-Students were satisfied with the learning environment at the DEU<br>-Experience at the DEU whipped (90%) the zeal in students to become Registered Nurses<br>-All students reported excellent relationships with DEU staff; they felt like part of the healthcare team.	Further studies should: Compare student perceptions of the clinical learning environment in traditional units with

S2 Table Characteristics of articles included in the scoping review

No	Author/ Year	Country	Title of the study	Design	Purpose of the study	Sample Size	Population	Name of strategy	Main Findings	Recommendations
									-68% of students learned more from DEU nurses than fellow students (32%)<br>-Students felt they were treated with respect at DEU<br>-Students had one-on-one engagements with nurses<br>-Students had the opportunity to ask questions which were answered satisfactorily (90%)<br>-Evidenced-based practice was evident at the DEU<br>-Students (74%) reported that patients' needs were a priority<br>-Students perceived that there was individualised patient care at the DEU<br>-There was value in professionalism<br>-A rewarding experience for DUE nurses<br>-Fostering of academia-practice partnership<br>-DEU fostered critical thinking	DEU and changing role of the faculty in this model.

S2 Table Characteristics of articles included in the scoping review

No	Author/ Year	Country	Title of the study	Design	Purpose of the study	Sample Size	Population	Name of strategy	Main Findings	Recommendations
									-All students indicated independent problem-solving was encouraged by the DEU<br><br>**-Perceived Challenges -**some students felt that Nurses at the DEU were not ready or committed and not trained, students and their DEU nurses were on different shifts, overcrowding of students at the DEU at certain times<br><br>**DEU Nurses**<br><br>-78% were satisfied with their involvement with students<br><br>-93% indicated that they met the needs of patients<br><br>-DEU helped nurses embrace team building and leadership<br><br>-Being a DEU nurse encouraged the nurses to seek further education<br><br>-DEU nurses were supported by faculty regarding how to handle students<br><br>Challenges of DEU Nurses	

S2 Table Characteristics of articles included in the scoping review

No	Author/ Year	Country	Title of the study	Design	Purpose of the study	Sample Size	Population	Name of strategy	Main Findings	Recommendations
									-Managing students as well as patients is a challenge<br><br>-Recognition of DEU nurses is limited to only those in charge of students<br><br>-The assignment of students to DEU is not well scheduled.<br><br>**Faculty**<br><br>-Involvement of DEU has lightened the clinical instructor role of faculty<br><br>-Faculty taught students about the role of DEU nurse<br><br>-Faculty mentioned its role to students.	
16	Rodriguez-Garcia et al. (2018)<br><br>(32)	Spain	Experiential learning in practice: an ethnographic study among nursing students and preceptors	Qualitative ethnographic	To explore the reflective dialogues and the reflective processes that occur between preceptors and nursing students and to examine how preceptors	undergraduate nursing students (n=27) Preceptors (n=15)	Undergraduate nursing students, Preceptors with more than 5 years' experience	Experiential learning	Professions perform their professional tasks based on previous experience.<br><br>The experience of preceptors helps facilitate the development of students' technical skills.<br><br>-Preceptors draw linkages between theory and practice, guiding	Current programs must distance themselves from learning by imitation to embrace significa

S2 Table Characteristics of articles included in the scoping review

No	Author/ Year	Country	Title of the study	Design	Purpose of the study	Sample Size	Population	Name of strategy	Main Findings	Recommendations
					make use of their expert knowledge to enhance students' experiential learning during clinical placements.				students' reflective abilities and enhancing their clinical learning.<br>-Students were given the opportunity to experiment with procedures, make mistakes, and learn from those mistakes.<br>-Students seek complete autonomy in the performance of procedures they have mastered. However, they need the presence/company of the expert.<br>-Preceptors reference students' mistakes, give the correct option, and ask students to ponder over the correct option to gain experiential knowledge.<br>-Preceptors use examples of everyday life to help students assimilate new concepts.<br>-Students use examples to demonstrate their understanding by describing their previous clinical encounters.<br>-Students were given professional roles to help them integrate	nt, experiential learning.

S2 Table Characteristics of articles included in the scoping review

No	Author/ Year	Country	Title of the study	Design	Purpose of the study	Sample Size	Population	Name of strategy	Main Findings	Recommendations
									knowledge acquired from preceptors.	
17	Farzi et al. (2018) (34)	Iran	Exploring the challenges of clinical education in nursing and strategies to improve it: a qualitative study	Qualitative method	To disclose the problems of clinical education in nursing and to provide the strategies to improve it.	Nursing students (n = 35), clinical educators (n =5)	Nursing educators and nursing students.	Simulation/communication/orientation	Strategies for improving clinical education of nursing<br>-The use of nursing education models and methods, such as the nursing process, simulation, and peer learning in clinical education.<br>- The nursing process enhances students' critical thinking<br>-Combining students of lower and higher classes during clinical sessions.<br>-Simulation<br>-Improvement of communication between faculty and practice to create a sincere atmosphere and alleviate fears and concerns of students.<br>-Holding orientation stage at the beginning of training<br>-The students believed that the clinical educator should introduce the lesson plan, the contents of the training course, and evaluation method during the orientation stage at the beginning of the clinical education so that the students know the contents of the course.	Not reported

S2 Table Characteristics of articles included in the scoping review

No	Author/ Year	Country	Title of the study	Design	Purpose of the study	Sample Size	Population	Name of strategy	Main Findings	Recommendations
									-Examine the cognitive and emotional domain of the student before clinical skills education, and if it is necessary, reforming measures should be taken.	
18	Dorcy et al. (2016) (51)	USA	From student to practicing oncology nurse: a novel collaboration to create a transition to practice program in ambulatory cancer care	Not reported	This article describes a sequential three-step creation of a DEU in an outpatient oncology unit at a large National Cancer Institute (NCI) designated comprehensive cancer centre.	Students (n = 54) Resident Nurses (n=8)	Students and Resident Nurses	Dedicated Education Unit Model	-Students who had clinical experiences at the DEU are highly employable<br>-Developed a resource pool for professional nurse acquisition<br>-Students with DEU experience are successful at state examinations<br>-Increase in job satisfaction to the preceptors.	Not reported
19	Lee et al. (2022) (54)	South Korea	Improving active collaborative e clinical learning through	A nested, mixed-methods design was used.	This study developed a mobile application for use in a pediatric	(n = 54)	Nursing Students	Mobile application	**Perceived Achievement of Learning Outcomes and Satisfaction with Clinical Practicum and mean scores**	Future research is needed to investigate the

S2 Table Characteristics of articles included in the scoping review

No	Author/ Year	Country	Title of the study	Design	Purpose of the study	Sample Size	Population	Name of strategy	Main Findings	Recommendations
			a mobile application for undergraduate nursing students		clinical environment to enhance student activity and self-regulated learning and to facilitate preceptor and nursing faculty collaboration and explored perceptions and experiences of the use of applications.				-The achievement level of learning outcomes was 4.1 out of 5<br>-Items regarding the nursing process application and understanding of nursing roles in multidisciplinary teams was 4.3 out of 5<br>-Items regarding effective communication with other healthcare providers and ethical and professional standards scored lowest was 3.9 out of 5.<br>-overall satisfaction with the practicum using the mobile application was 4.3 out of 5<br>- Students were most satisfied with the preceptors (4.5 out of 5)<br>-the mean score for the mobile application and clinical sites was 4.4 out of 5, followed by practicum content with 4.3 out of 5.<br>**Experience of Participants Regarding Their Use of the Mobile Application**<br>Theme 1: Facilitated Collaborative	effects of the application in clinical practice on long-term learning outcomes by validated scales such as nursing competence and self-efficacy.

S2 Table Characteristics of articles included in the scoping review

No	Author/ Year	Country	Title of the study	Design	Purpose of the study	Sample Size	Population	Name of strategy	Main Findings	Recommendations
									Learning Through Real-Time Interactions and Communication<br>-Participants had more opportunities to interact with clinical preceptors through the mobile application.<br>-Participants could ask questions in the clinical unit using the application and receive constructive feedback more promptly from the preceptors because both shared the same materials provided in the application.<br>-The application allowed preceptors to identify participants' needs, misunderstandings, and required learning outcomes timeously.<br>-Preceptors could use the same materials included in the mobile application when demonstrating clinical skills<br>-Participants had opportunities to discuss what they learned with their peers and faculty and to get prompt feedback.<br>-Students shared useful resources with preceptor.	

S2 Table Characteristics of articles included in the scoping review

No	Author/ Year	Country	Title of the study	Design	Purpose of the study	Sample Size	Population	Name of strategy	Main Findings	Recommendations
									-Preceptors used the checklist and asked about students' learning contents<br>-Preceptor and students used the same materials helped students' learning.<br>**Theme 2: Engagement in Active Learning Through a Mobile Application**<br>-Access to the learning process based on their situations (pace and need)<br>-Mobile application-supported access to learning anytime and anywhere. They used the learning materials depending on their situation. In addition,<br>-students used educational resources when they prepared for clinical practice and reviewed their learning over the course.<br>-convenient to use<br>-video clips or other educational materials helped to practice nursing skills or acquire nursing knowledge at any time.<br>-educational materials are useful in providing education to patients.<br>-Student-centered active learning	

S2 Table Characteristics of articles included in the scoping review

No	Author/ Year	Country	Title of the study	Design	Purpose of the study	Sample Size	Population	Name of strategy	Main Findings	Recommendations
									- Participants could recognize learning gaps in clinical settings.<br>- The application had a checklist that promoted self-regulated learning based on participants' individualized progress.<br>-Application keeps students engaged in learning in the clinical environment.<br>**Theme 3: A Useful Tool for Enhancing Critical Thinking**<br>-Students are able to execute the nursing process more dynamically and timeously.<br>- Real-time recording of their patient progress<br>-Improves critical thinking.<br>-Limited space for the nursing process on application leads to critically prioritizing data and nursing problems<br>-Prompt completion of the nursing process during the clinical practice.<br>**Theme 4: The Curate's Egg Nature of the Application**<br>-Enhanced learning outcomes	

S2 Table Characteristics of articles included in the scoping review

No	Author/ Year	Country	Title of the study	Design	Purpose of the study	Sample Size	Population	Name of strategy	Main Findings	Recommendations
									-Helped manage work efficiently and saved time and paper.<br><br>-Some participants preferred paper-based material because standardized formats in the application might hinder students' expanded thinking in the learning process.<br><br>**Challenges**<br>-Difficulties in adopting the new technology, technical errors, including data loss, charging issues, and unstable network connectivity.<br>-Participants assessed patients and recorded in predetermined and limited mobile application format leading to missing of other nursing problems.<br><br>-Unable to think beyond the application<br><br>-It took time to adjust to this new technology<br><br>-Cases of anger when encountering technical errors.	

S2 Table Characteristics of articles included in the scoping review

No	Author/ Year	Country	Title of the study	Design	Purpose of the study	Sample Size	Population	Name of strategy	Main Findings	Recommendations
20	Pimmer et al. (2018) (53)	Nigeria	Instant messaging and nursing students' clinical learning experience	Quantitative	To investigate the use of WhatsApp and its correlation with a number of socio-professional indicators.	(n=196, 25 men and 171 women)	Final year students in five Schools of Nursing in South-West, Nigeria. Nursing students	Instant messaging	-Students used WhatsApp relatively frequently<br>-Students reported high levels of social capital, identity development, placement satisfaction and lower levels of professional isolation.<br>-Positive correlation between the students' WhatsApp use during placement and professional identity and placement satisfaction<br>-Negative association between WhatsApp use during placement and students' feeling of professional isolation<br>-WhatsApp supports communication with students and nurses<br>-The perceived ease of use is significantly associated with WhatsApp use during placement	Future research should corroborate the findings of this study, but to develop a better understanding of the dynamics of mobile instant messaging (MIM) use, especially of other mediating factors that can play into the use of MIM

S2 Table Characteristics of articles included in the scoping review

**No**	**Author/ Year**	**Country**	**Title of the study**	**Design**	**Purpose of the study**	**Sample Size**	**Population**	**Name of strategy**	**Main Findings**	**Recommendations**
										in clinical learning contexts, such as learners' personal traits or the number and quality of pre-existing relationships.
21	Strout et al. (2017) (49)	USA	Interprofessional mass casualty incident simulation design protocol to prepare prelicensure nursing students to respond to a disaster	Not reported	To prepare prelicensure nursing students to respond effectively to disasters.	Senior nursing students (n=30), and registered nurses (n=15).	Senior nursing students and RNs were enrolled.	Interprofessional mass casualty incident simulation	Most of the students (96%) indicated increased confidence in responding to a disaster. -All of the participants' overall event rating was 94.25 (n = 40).	Not reported

S2 Table Characteristics of articles included in the scoping review

No	Author/ Year	Country	Title of the study	Design	Purpose of the study	Sample Size	Population	Name of strategy	Main Findings	Recommendations
22	Lait et al. (2011) (65)	Canada	Interprofessional mentoring: enhancing students' clinical learning	Qualitative	To describe a study in which we implemented IP mentoring between staff and students at a number of clinical sites and evaluated its success.	Students (n=34). Providers (n=52) across the five sites.	Nursing Students and providers across the five sites.	Interprofessional mentoring	Other care providers play the role of mentors by connecting students with staff across disciplines.<br>Interaction with other professionals is enhanced.<br>Students view how other care providers do their work/increase their knowledge of the roles of other providers.<br>Students take part in team meetings, conferences, and consultations.<br>Enhances learning beyond the classroom.<br>It helps student nurses to understand that their roles involve working with others.	Not reported
23	Tang and Chang (2019) (63)	Hong Kong	Learning experience of nursing students in a clinical partnership model: An exploratory	Qualitative	To explore students' clinical learning experiences using the clinical	(n=28)	Nursing students	Clinical partnership Model	-Familiarity of clinical teachers to the ward helped the students to adapt to the clinical environment leading to less tension among students and minimal risk of committing mistakes.	Future studies can compare students' clinical competence under

S2 Table Characteristics of articles included in the scoping review

No	Author/ Year	Country	Title of the study	Design	Purpose of the study	Sample Size	Population	Name of strategy	Main Findings	Recommendations
			qualitative analysis		partnership model.				-Other staff were willing to teach students when their clinical teachers were absent.<br>-The approach supports communication.<br>-Clinical teachers provided more learning opportunities for students than school teachers.<br>-Exposure of students to more clinical tasks.<br>-Students were taught how to blend theoretical knowledge with clinical practice.<br>-Students learned from clinical practice that knowledge is flexible, unlike textbook fixed knowledge.<br>-Students learned the professional roles of nurses<br>-Model fostered good collaboration between clinical teachers and faculty.<br>-Teachers from the school monitored students at the clinical site, providing feedback and sharing	various clinical teaching models.

S2 Table Characteristics of articles included in the scoping review

No	Author/ Year	Country	Title of the study	Design	Purpose of the study	Sample Size	Population	Name of strategy	Main Findings	Recommendations
									their experiences to facilitate learning	
24	Day-Black (2015)<br>(29)	USA	Minorities in nursing education: using Smartphones	Not reported	To present a discussion on current African-American smartphone practices, and its impact on minority nursing students and implications for HBCU nursing programs.	Not reported	Nursing students at Coppin State University (CSU).	Smartphones	-Smartphone support anytime and anywhere learning<br>-Students can download useful resources (drug references, medical information, drug calculation/dosing, electronic version of textbooks) or applications to support learning<br>-Smartphones promote patient safety<br>-Slow adoption of smartphones into the nursing curriculum<br>**Factors hindering adoption**<br>-Smartphones<br>-Lack of nursing faculty development<br>-Faculty's resistance to change<br>-Lack of role-modeling by nursing faculty<br>**Major reasons for reluctance to develop new technology pedagogy**	Not reported

S2 Table Characteristics of articles included in the scoping review

No	Author/ Year	Country	Title of the study	Design	Purpose of the study	Sample Size	Population	Name of strategy	Main Findings	Recommendations
									-smartphones are distractions in the classroom<br>-more thoughts and strategic planning are required among nursing faculty of successful integration	
25	Bϕ et al. (2021)<br>(41)	Tanzania and Madagascar	Nursing students' experiences with simulation-based education as a pedagogic method in low-resource settings: a mixed-method study	Mixed-method design	To address nursing students' experiences with simulation-based education as a pedagogic method within the topic of emergency nursing.	Third-year Nursing students from Tanzania (n=53) first-year students from Madagascar (n=46)	Third-year nursing students from Tanzania and first-year students from Madagascar	Simulation-based education	**Quantitative**<br>Students rated scores above 4 on a 5-point Likert scale on the following<br>-simulation design elements<br>-Educational practices<br>-Students' satisfaction<br>-Students' self-confidence in learning<br>-Active learning<br>-Collaboration<br>-Diverse ways of learning<br>-High expectations<br>**Qualitative**	Further research is necessary to explore whether the students can transfer their knowledge into clinical practice.

S2 Table Characteristics of articles included in the scoping review

No	Author/ Year	Country	Title of the study	Design	Purpose of the study	Sample Size	Population	Name of strategy	Main Findings	Recommendations
									-Prepares students to become competent and confident<br>-Simulation supports active learning<br>-Anxiety among students playing the scenarios, anxiety higher with teachers than with fellow students.	
26	Nielsen et al.<br>(2013) (28)	USA	Preparing nursing students for the future: An innovative approach to clinical education	Not reported	To describe the need for new approaches to clinical education, reviews the Oregon Consortium for Nursing Education (OCNE) curriculum and its associated clinical education model, then provides specific examples of innovative	Not provided	Nurse educators	Oregon clinical educational model/Curriculum review	Curriculum consideration<br>-Deliberate sequencing /arrangement of increasingly complex learning experiences in the following order:<br>-Intervention skill-based<br>-Case-based<br>-Concept-based<br>-Focused direct client care and<br>-Integrative experiences	Not reported

S2 Table Characteristics of articles included in the scoping review

No	Author/ Year	Country	Title of the study	Design	Purpose of the study	Sample Size	Population	Name of strategy	Main Findings	Recommendations
					ways that nurse learning, with a particular emphasis on case-based, concept-based and integrative clinical experiences.					
27	Liaw et al., (2012) (42)	Singapore	Recognizing, responding to and reporting patient deterioration: Transferring simulation learning to patient care settings	Qualitative	To explore nursing students' experiences on how a simulation programme prepared them to transfer their clinical performance, in their encounters with deteriorating patients in wards.	(n=15)	Nursing students	Simulation	Factors influencing students' abilities to transfer learning from simulation to real patient care setting<br>-memory (retrieval of knowledge from memory, retention of learning in long-term memory)<br>-mnemonics as transfer tools (for patient assessment, for reporting)<br>-recognizing similar situations (for identifying problems, for initiating immediate intervention)<br>-emotional response (feeling of calm, feeling of stress)	Further development of the simulation programme should: Address the potential emotional issues.<br>Include of real human patients

S2 Table Characteristics of articles included in the scoping review

No	Author/ Year	Country	Title of the study	Design	Purpose of the study	Sample Size	Population	Name of strategy	Main Findings	Recommendations
									Strategies to enhance simulation programme to facilitate transfer of learning<br>-Realism (replacing mannequin with simulated patient, more variations in simulation scenario)<br>-Self-directed learning (revision resources)	within the simulation training sessions.<br>Combine real people (simulated patients) with inanimate simulators that allows the learners to engage in patient interaction while performing a task.<br>Include multime

S2 Table Characteristics of articles included in the scoping review

No	Author/ Year	Country	Title of the study	Design	Purpose of the study	Sample Size	Population	Name of strategy	Main Findings	Recommendations
										dia resources that could demonstrate clinical performances to supplement their simulation learning.
28	Lee and Lapum<br>(2018) (40)	Canada	Student-guided, theme-based post-clinical conference to enhance student involvement in clinical learning.	Not reported	To describe a student-driven, theme-based approach in conducting PCCs	(n=15)	Students in their final term of a 4-year baccalaureate nursing program	Post-clinical conference	-Active participation of students.<br>-Reflective practice.<br>-Discussions during the conference revealed students blending clinical experience with theoretical knowledge.<br>-Students felt connected to the program and peers in the post-clinical conference.<br>-Common interest was identified among students.<br>-Students learned from each other.	Future research should study the effect of post-clinical conferences on learning outcomes, clinical practice, and student

S2 Table Characteristics of articles included in the scoping review

No	Author/ Year	Country	Title of the study	Design	Purpose of the study	Sample Size	Population	Name of strategy	Main Findings	Recommendations
										involvement and engagement.
29	Cheraghi et al. (2021) (61)	Iran	The effect of peer support approach on communication skills of nursing students in paediatric clinical setting	Quasi - experimental study with a pre and post-test design with intervention and control groups.	To determine the effect of peer support approach to the communication skills of nursing students when interacting with hospitalized children and their parents in a paediatric setting of a large tertiary hospital in Hamadan City, Iran.	Nursing students (n = 103) intervention (n =51), and control group (n =52).	Final-year undergraduate students in a four-year Bachelor of Nursing programme.	Peer support approach	The peer support approach promoted the communication skills of undergraduate nursing students	Further studies are needed to assess the perceived outcomes of the collaboration of students using the peer support approach. Clinical educator perceptions of the clinical peer partnership are

S2 Table Characteristics of articles included in the scoping review

No	Author/ Year	Country	Title of the study	Design	Purpose of the study	Sample Size	Population	Name of strategy	Main Findings	Recommendations
										also worth exploring.
30	Hwang et al. (2022) (57)	Taiwan	The effectiveness of the virtual patient-based social learning approach in undergraduate nursing education: a quasi-experimental study	A quasi-experiment	To use a VP interactive system to provide an innovative education model for nursing students and demonstrate the impacts of the system from different perspectives of educational objectives by referring to the social learning theory	One class (n= 20) was assigned to the experimental group adopting the VP-based social learning approach. The other class (n = 20) was the control group learning with the convent	The participants were two classes of junior students who took the physical assessment course in a nursing university in northern Taiwan.	Virtual patient (VP)-based social learning approach	Experimental results indicate that using VP for learning can enhance students' learning achievements, self-efficacy, and communication skills<br>-students believed that learning with VP makes learning more enjoyable	Future studies should (1) investigate the factors that affect students' learning effectiveness, it could be necessary to conduct studies using the VP-based social learning approach with larger

S2 Table Characteristics of articles included in the scoping review

No	Author/ Year	Country	Title of the study	Design	Purpose of the study	Sample Size	Population	Name of strategy	Main Findings	Recommendations
						ional learning approach.				sample groups. (2) Examine students' interactive content and behavioural patterns during the learning process, which could provide additional evidence to explain why the VP-based social learning approach is more

S2 Table Characteristics of articles included in the scoping review

No	Author/ Year	Country	Title of the study	Design	Purpose of the study	Sample Size	Population	Name of strategy	Main Findings	Recommendations
										effective than the conventional one. (3) the effectiveness of the VP-based social learning approach should be explored from different angles. For example, in investigating learners' critical thinking performance (4) researchers can

S2 Table Characteristics of articles included in the scoping review

No	Author/ Year	Country	Title of the study	Design	Purpose of the study	Sample Size	Population	Name of strategy	Main Findings	Recommendations
										apply the VP-based social learning approach to other nursing education programs to further examine the effectiveness of this approach.
31	Ulvund and Mordal (2017)<br>(66)	Ethiopia	The impact of short-term clinical placement in a developing country on nursing students: a qualitative	Qualitative	To investigate how short-term international clinical placement impacted Norwegian nursing students' development	(n=18)	Nursing students in the Norwegian University College Bachelor's	International clinical placement	-Creation of cultural awareness and adjustment<br>-Igniting the sense of compassion and cohesion among students<br>-The realities of cultural shock<br>-Expansion of personal standpoints<br>-Knowledge development	Further research is recommended to develop insights into how the process continue

S2 Table Characteristics of articles included in the scoping review

No	Author/ Year	Country	Title of the study	Design	Purpose of the study	Sample Size	Population	Name of strategy	Main Findings	Recommendations
			descriptive study		of cultural competency.		Programme.		-Appreciation of different working conditions<br>-The sense to contribute to the knowledge base and emotional well-being of others.<br>The academic knowledge base of nurses from the two different countries was not widely variant<br>-Patient relatives were prominent in-patient care<br>-Professionals in the host county lacked empathy for patients and relatives	s after the students have returned home and how the nursing programme can support this process.
32	Watson (2015) (64)	USA	The influence of participating in an international clinical experience during baccalaureate nursing education	Qualitative	To describe newly prepared baccalaureate nurses' experiences on the development of the competency for	(n=8)	The population of this study had two distinctions. 1. Baccalaureate-prepared	International clinical experience	-Students felt valued and respected by other healthcare team members.<br>-Students felt their activities (e.g. checking of vital signs) mattered to the healthcare team<br>-Students had the opportunity to conduct ward rounds with doctors, unlike in their home countries.	Future research should: advance the body of knowledge on interprofessional, internati

S2 Table Characteristics of articles included in the scoping review

**No**	**Author/ Year**	**Country**	**Title of the study**	**Design**	**Purpose of the study**	**Sample Size**	**Population**	**Name of strategy**	**Main Findings**	**Recommendations**
			on interprofessional collaboration and teamwork for new registered nurses		interprofessional collaboration and teamwork after participating in an international clinical experience during their prelicensure nursing education.		nurses in their first year of employment as a registered nurse. 2. These nurses participated in an interprofessional, international clinical field experience during their undergraduate nursing education program.		-Students reported healthcare team had interprofessional cohesiveness due to a shared purpose of rendering care to underserved communities. -Students developed effective communicative skills (openly and confidently) as a result of exposure to the interprofessional team -Developed professional, respectful relationships -Increased self-confidence -Enhanced professional identity and understanding of professional roles and responsibilities. -Experience of autonomy within their professional scope (their professional judgements were valued). -Improvement in nursing skills -learned interprofessional collaboration and teamwork -Appreciation of abundance and accessibility	onal clinical experience, investigate the long-term influence on different healthcare professionals, conduct a longitudinal study that explores the impact of an international clinical experience more than 2

S2 Table Characteristics of articles included in the scoping review

No	Author/ Year	Country	Title of the study	Design	Purpose of the study	Sample Size	Population	Name of strategy	Main Findings	Recommendations
									-Understanding the realities of others from/working in less endowed settings<br>-Shaping their civic responsibilities<br>-Cultural sensitivity and awareness (being less judgemental, open-mindedness)<br>-Igniting the sense of helping others.	years past the experience, more along the timeframe of 5 years post-the experience.
33	Botha et al. (2021) (55)	South Africa	The reality of virtual reality at a South African university during the COVID-19 pandemic	Not reported	To develop the desktop-based virtual reality platform aimed at improving student access to the virtual reality learning experience.	(n= 34)	Undergraduate nursing students in their third or fourth year.	Desktop-based virtual reality platform	-The desktop-based virtual reality platform afforded students in quarantine or isolation an opportunity to continue learning.<br>-The students continued learning with limited physical contact, thus reducing the chances of spreading COVID-19.<br>-The virtual clinical learning platform can be re-used at the student's convenience.<br>-Students may only develop clinical reasoning skills as their decisions on the care of the virtual patient may directly result in a simulated	Not reported

S2 Table Characteristics of articles included in the scoping review

No	Author/ Year	Country	Title of the study	Design	Purpose of the study	Sample Size	Population	Name of strategy	Main Findings	Recommendations
									physiological response.<br>**Challenges**<br>-students will not develop any nursing-related psychomotor skills from this platform.<br>-The rapid development of the platform may result in errors.<br>-Use of students' internet resources might limit the platform's utility.<br>-Time and support commitment. A dedicated support person is needed to assist with queries.	
34	Coffey et al. (2022) (44)	Australia	Final year undergraduate nursing students' experience of high-fidelity simulation: results of a survey	An exploratory-descriptive design.	To describe undergraduate nursing students' high-fidelity simulation experience in an Australian context using the validated Satisfaction with Simulation Experience Scale (SSES).	(n=288)	Undergraduate nursing students	High-fidelity simulation	The majority of students strongly agreed that high-fidelity simulation enhanced their learning (98.9%), developed their clinical reasoning skills (97.2%) and clinical decision-making ability (96.9%), and<br>that simulation was a valuable learning experience (99.3%).	Not reported

S2 Table Characteristics of articles included in the scoping review

No	Author/ Year	Country	Title of the study	Design	Purpose of the study	Sample Size	Population	Name of strategy	Main Findings	Recommendations
35	Kacalek et al. (2022) (46)	USA	The Clinical Pause: an augmented approach to simulation debriefing in Nursing Education	Not reported	Not reported	(n=24)	Nursing students	High-Fidelity Simulation	-Students indicated that clinical pause facilitated timely knowledge construction, contextual thinking, and clinical judgment.<br>-Faculty perceived the clinical pause approach provides timely feedback, reflection-in-action, and opportunities to enhance students' thinking skills.<br>-The timing of the clinical pause promoted rich discussion and robust learning based on recent recall rather than remote recollection.	Not reported
36	Adongo et al. (2023) (60)	Uganda	"No PBL is better than online PBL": qualitative exploration regarding the perceived	Qualitative	To explore perceptions of students regarding the benefits of online PBL and its associated impact on the	Nursing students (n=14), and medicine	Bachelor of Nursing students, and Medical students	Online problem-based learning (PBL)	-Students perceived that learning was less online compared to face-to-face sessions because of reduced learner engagement, concentration, motivation, peer-to-peer learning, and limited opportunities for practical sessions.	Not reported

S2 Table Characteristics of articles included in the scoping review

No	Author/ Year	Country	Title of the study	Design	Purpose of the study	Sample Size	Population	Name of strategy	Main Findings	Recommendations
			impact of online problem-based learning on nursing and medical students' learning during COVID-I9 lockdown		learning experience of students in Busitema University.	students (n=20)			-Online learning was thought to increase students' workload in the form of several assessments, which was thought to reduce learning. -Online tutorials were perceived to reduce the acquisition of soft skills like confidence, communication, leadership, and practical or clinical skills. - It allowed continued learning during the lockdown, -Noted to be flexible - Enhanced self-drive and opportunity for work, solve infrastructure problems, and protect them from COVID-19 infection	
37	Laugaland et al. (2022) (59)	Norway	Co-creating digital educational resources to enhance quality in student nurses' clinical education	A co-design methodology, an approach that builds on the	To report the methodological development process of an interactive digital educational resource	Student nurses (n=10), nurse educators (n=9), register	Student nurses, nurse educators, registered nurses	Development of digital educational resources using design thinking	Phase 1: in the empathize phase, the researcher strives to understand and gain insights into stakeholders' needs. Phase 2: the define phase aims to determine specific, meaningful challenges to be addressed in the	Not reported

S2 Table Characteristics of articles included in the scoping review

No	Author/ Year	Country	Title of the study	Design	Purpose of the study	Sample Size	Population	Name of strategy	Main Findings	Recommendations
			in nursing homes: Report of a co-creative process	principles of participatory design	targeted to enhance the quality of clinical education in nursing homes.	ed nurse (n=17)		framework	interactive digital educational resource<br>Phase 3: Ideation phase: joint workshop with all stakeholders.<br>Phase 4: Prototyping a digital educational resource to enhance mentorship practices.<br>Phase 5: Pilot testing and evaluation to refine and spark new ideas.<br>Feedback is solicited from users about the prototype that has been created.<br>Phase 6: Refine prototype and solutions (educational resource's design, content, and functionality).<br>Phase 7: Pilot testing and evaluation of the digital educational resource targeting all stakeholder groups	
38	Panepucci et al. (2022) (47)	USA	Learning with laughter: implementing	Not reported	To describe a remediation strategy focusing on the needs of	(n=99)	Prelicensure Nursing students	Virtual simulation	-Students agreed the simulation was effective in all the areas (pre-briefing, learning, confidence, and debriefing).	Not reported

S2 Table Characteristics of articles included in the scoping review

No	Author/ Year	Country	Title of the study	Design	Purpose of the study	Sample Size	Population	Name of strategy	Main Findings	Recommendations
			engaging virtual simulation during the COVID-19 Pandemic		aggregates and using engaging simulation.				-Students indicated the activity was a fun and interactive way to enhance learning.<br><br>-Students found the stopping points helpful and liked interacting as a group through real-time engagement with the educators.<br><br>-Stopping points allowed them to project their thoughts and opinions, practice prioritization skills, and make decisions in real-time before the simulation evolved.<br><br>-Students determined appropriate assessments and interventions the nurse should implement and observe the results of the nurse's actions through coaching during each pause.<br><br>-Students enjoyed observing the simulation and seeing professionals enact the roles.	

S2 Table Characteristics of articles included in the scoping review

No	Author/ Year	Country	Title of the study	Design	Purpose of the study	Sample Size	Population	Name of strategy	Main Findings	Recommendations
									faculty and simulation educators provided immediate feedback to students on their decision-making	
39	Sundal and Ulvund, (2022)<br>(67)	Ethiopia	Experiences after international clinical placement as nursing students in a paediatric ward	Qualitative study, a hermeneutic, phenomenological approach	To investigate nurses' experiences after participating in an international clinical<br>placement programme as nursing students while staying for one to three weeks in a paediatric ward.	(n=8)	Nurses who participated in ICP programmes as part of their bachelor's degree programme	International clinical placement	-Meetings with children's destinies as patients were overwhelming<br>-Being an observer provided insights into and an overview of paediatric nursing<br>-Limited access to the resources required<br>-Nurses had a different role, and parents had extended caring roles.<br>-Participants gained knowledge of children as patients at a hospital and also gained knowledge of<br>the parents' and nurses' roles and the treatment methods of various diseases that are relevant to paediatric<br>nursing.<br>-Participants gained cultural knowledge, awareness and sensitivity, given the contrasting situation in their home country.	Not reported

S2 Table Characteristics of articles included in the scoping review

No	Author/ Year	Country	Title of the study	Design	Purpose of the study	Sample Size	Population	Name of strategy	Main Findings	Recommendations
40	Roop, (2022) (45)	USA	Replacing the paediatric traditional clinical experience with high-fidelity simulation in an associate degree nursing program	Mixed methodology action research	To compare and assess the traditional clinical experience with high-fidelity simulation and evaluate the effectiveness of high-fidelity simulation as a replacement for the traditional clinical experience.	(n=26)	Nursing students	High-Fidelity Simulation	- High-fidelity simulation met the learning needs of the nursing students better than the two other clinical learning environments.<br>-The screen-based simulation (virtual simulation) environment met the student learning needs in the critical thinking subscale better than the other two clinical learning environments.<br>-The traditional clinical environment met the student learning needs in the teaching-learning dyad subscale better than the two other clinical learning environments.<br>-The high-fidelity face-to-face simulated clinical environment met the student learning needs in the holism subscale better than the two other clinical learning environments.<br>-The high-fidelity face-to-face simulated clinical environment had a<br>shortcoming identified in the area of pre-briefing	- Introduce open lab time before High Fidelity Simulation<br>-Provide adequate time for pre-briefing before high-fidelity simulation<br>-Utilise the clinical learning environment comparison

S2 Table Characteristics of articles included in the scoping review

No	Author/ Year	Country	Title of the study	Design	Purpose of the study	Sample Size	Population	Name of strategy	Main Findings	Recommendations
									-High-fidelity simulation utilised fewer nursing faculty while educating the same number of nursing students for the required clinical hours in a paediatric traditional clinical experience.	survey (CLECS) 2.0 tool as the evaluation tool in the nursing of families with children course and other nursing courses
